# The nonsteroidal anti-inflammatory drug meclofenamate mitigates kainic acid–induced seizures via TRPM4 inhibition

**DOI:** 10.1093/braincomms/fcaf229

**Published:** 2025-06-12

**Authors:** Erzsébet Kövesdi, Laura Mundrucz, Attila Gyéresi, Máté Deák, Balázs Gaszner, Andy Pironet, Cecília Szekeres-Paraczky, Zsófia Maglóczky, Péter Gombás, Rudi Vennekens, Viktória Kormos, Miklós Kecskés

**Affiliations:** Institute of Physiology, Medical School, University of Pécs, Pécs H-7624, Hungary; Institute of Physiology, Medical School, University of Pécs, Pécs H-7624, Hungary; Institute of Physiology, Medical School, University of Pécs, Pécs H-7624, Hungary; Institute of Physiology, Medical School, University of Pécs, Pécs H-7624, Hungary; Department of Anatomy, Medical School and Research Group for Mood Disorders, Centre for Neuroscience, University of Pécs, Pécs H-7624, Hungary; Laboratory of Ion Channel Research, Biomedical Sciences Group, Department of Cellular and Molecular Medicine, VIB-KU Leuven Center for Brain & Disease Research, KU Leuven, Leuven 3000, Belgium; Human Brain Research Laboratory, HUN-REN Institute of Experimental Medicine, Budapest H-1083, Hungary; Szentágothai János Doctoral School of Neuroscience, Semmelweis University, Budapest 1085, Hungary; Human Brain Research Laboratory, HUN-REN Institute of Experimental Medicine, Budapest H-1083, Hungary; Department of Pathology, St. Borbála Hospital, Tatabánya H-2800, Hungary; Laboratory of Ion Channel Research, Biomedical Sciences Group, Department of Cellular and Molecular Medicine, VIB-KU Leuven Center for Brain & Disease Research, KU Leuven, Leuven 3000, Belgium; Department of Pharmacology and Pharmacotherapy, Centre for Neuroscience, Medical School, University of Pécs, Pécs H-7624, Hungary; Institute of Physiology, Medical School, University of Pécs, Pécs H-7624, Hungary

**Keywords:** TRPM4, meclofenamate, seizure, mossy cell, hippocampus

## Abstract

Transient receptor potential melastatin 4 (TRPM4) is a Ca^2+^-activated non-selective cation channel that regulates various physiological functions of excitable cells. In accordance with our previous findings, TRPM4 is known to be present and to be functionally active in hilar mossy cells where it controls seizure susceptibility. With the help of *in vivo* and *in vitro* electrophysiological and histological experiments, we investigated the effect of TRPM4 channel inhibition upon kainic acid–induced seizures. In this study, we present that *in vivo* administration of meclofenamate a novel blocker of TRPM4 before kainic acid injection reduces both seizure frequency and duration in mice. Furthermore, our findings reveal that meclofenamate treatment prior to kainic acid injection selectively reduced mossy cell loss in the ventral hippocampus. Interestingly, we observed elevated expression of TRPM4 in mossy cells of the ventral hippocampus highlighting the heterogenity of these neurons in the hippocampus. In addition, patch clamp recordings revealed that meclofenamate modulates both the spontaneous activity and the action potential dynamics of mossy cells. Lastly, we revealed the presence of *TRPM4* transcript in human mossy cells. Altogether, these findings suggest that pharmacological inhibition of TRPM4 may reduce seizure frequency thus possibly protect mossy cells.

## Teaser

Kövesdi *et al*. report that the nonsteroidal anti-inflammatory drug meclofenamate reduces seizure severity after systemic kainic acid injection *via* blocking transient receptor potential melastatin channel 4 in hippocampal mossy cells.

## Graphical Abstract

**Graphical Abstract:**
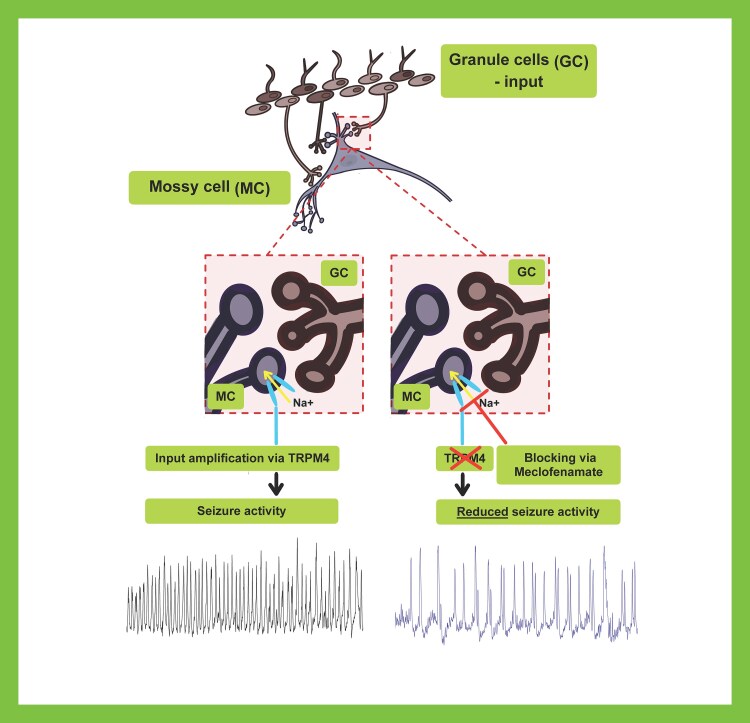

## Introduction

The dentate gyrus (DG) is considered as a gateway to the hippocampus as it filters the incoming information from the cortex.^1^ The DG plays a vital function in a number of cognitive tasks, such as learning, memory formation and anxiety.^2^ Moreover, the DG is associated with the pathophysiology of several neuro-psychiatric conditions, such as medial temporal lobe epilepsy.^3^ Based on these diverse functions, it is particularly important to understand how the neuronal subpopulations within the DG ensure its normal functions and how these are disrupted in disease. A long-standing question in the field is the role of a specific cell type called mossy cells (MC) both in the healthy and diseased DG.

MCs are located exclusively within the hilus of the DG, with projections to the ipsilateral and contralateral DG.^3^ MCs innervate both glutamatergic granule cells and inhibitory GABAergic interneurons.^4-6^ Thus, they can either directly excite or -through GABAergic interneurons- indirectly inhibit the granule cells.^7^ Despite their possible role in both DG excitation and inhibition, current data indicate that under physiological conditions MCs exert a net inhibitory effect on DG.^8^ However, in certain conditions, MC excitation of granule cells can increase. For instance, in status epilepticus (SE), the inhibitory function of MCs can turn into a strong excitation within the DG.^9^ Thus, inhibiting MCs during SE can reduce seizure severity.^9^

In our previous study, we demonstrated that transient receptor potential melastatin 4 (TRPM4) a member of the transient receptor potential family of ion channels^10^ is expressed in hilar MCs, and as a Ca^2+^-activated cation channel regulates their intrinsic electrophysiological properties.^11^ In addition, we showed that TRPM4 plays a role in MC death following SE thus altering seizure susceptibility, as well as epilepsy-related memory impairments. Based on our previous results, we hypothesized that the pharmacological blocking of TRPM4 may stand as a basis for novel seizure management strategies. However, up until now, the lack of potent and *in vivo* applicable TRPM4 blockers limited our possibilities to test the physiological effect of TRPM4 inhibition during seizures. Especially, because the most often used TRPM4 blocker 9-phenantrol has highly unfavourable pharmacokinetic properties,^12^ and it is only partially selective.^13,14^ Similarly, flufenamic acid is reported to block TRPM4; however, because of its frequent adverse effects and poor water solubility, it is not ideal candidate for *in vivo* experiments.^15^ CBA is introduced recently as a potent and selective small molecule TRPM4 blocker; however, its *in vivo* applicability is not yet proved.^16^ Recently, we identified that the nonsteroidal anti-inflammatory drug meclofenamate is a potent antagonist of TRPM4 and applicable *in vivo.*^17^ Meclofenamate is used to treat muscular pain and arthritis. As a nonsteroidal anti-inflammatory drug, it inhibits cyclooxygenases and therefore prevents prostaglandin synthesis. The half-life of meclofenamate ranges between 1 and 5 h when taken orally.^18^

Here, we show that the *in vivo* administration of meclofenamate reduced the length of epileptic seizures and delayed the first behavioural seizures following systemic kainic acid (KA) injection in mice. Furthermore, MC loss often seen after SE was reduced in the ventral region of the hippocampus—where TRPM4 is more abundantly expressed—upon meclofenamate treatment. These effects are most likely TRPM4 dependent since the application of meclofenamate in *Trpm4^−/−^* mice had no protective effect in KA-induced seizures. Of note, *in vitro* application of meclofenamate modified the intrinsic electrophysiological properties of MCs and reduced their excitability in epileptic conditions. Finally, we showed that TRPM4 is expressed in human MCs as well, highlighting the translational aspects of our findings.

## Materials and methods

### Experimental animals

We used 8–10 weeks old male C57BL/6N mice [wild type (WT)] and age-matched *Trpm4^−/−^* mice. Female mice were not used in the study because of the known sex differences in SE induction and mortality.^19^ The *Trpm4*^−/−^ mouse line generated earlier^20^ was bred in homozygous form, and after every fourth generation, mice were backcrossed into the C57BL/6N background, which served as the WT. The following primer pairs were used for genotyping: WT and knock out (KO) reverse 5′-gtt tga tgt ctc ctt cag tcg-3′; KO forward: 5′-gag ttc ctg tcc tcc taa agg-3′; WT forward: 5′-acc tac agg aaa cct cgg gg-3′. Moreover, naïve male C57BL/6N mice (*n* = 4) were used for the evaluation of the *Trpm4* mRNA expression in the hippocampus. Mice were housed in cages (3–5 mouse/cage) under a 12 h light/12 h dark cycle with free access to water and standard rodent food. All procedures were performed according to the European Community Council Directive and approved by the local ethics committee (Ethics Committee on Animal Research of Pécs, Hungary, BA02/2000-49/2023).

### Implantation of electrodes for EEG measurement

The EEG electrodes consisting of two recording electrodes (tungsten, 0.05 mm, insulated, GoodFellow) a reference and a ground wire were prepared individually. The wires were soldered to a custom-made six-pin connector suitable to be connected with the preamplifier. For the electrode implantation, mice were placed under deep anaesthesia by inhalation of oxygen containing isoflurane (5% induction, 2% maintenance). The head of the mice was shaved, and part of the skin was removed. The stereotaxic location of the craniotomy for the electrodes was the following: recording electrodes into the hippocampus: AP: −2.00 mm, ML: 1.05 mm (right and left); reference electrode: AP: −2.00 mm, ML: 2.05 mm to the left side. First, using a dental drill, three craniotomies were made (each smaller than 1 mm in diameter) in accordance with the abovementioned locations so that the EEG electrodes could be implanted (DV: −1.6 mm). The fourth electrode (ground electrode) was inserted under the skin. Finally, the six-pin connector was fixed to the skull with dental cement. After the surgery, all mice were transferred back into their home cages (three mice/cage) and treated with carprofen (s.c. 5 mg/kg) for pain relief. During the following 3 days, the animals were kept under postoperative observation.

### Treatments

Before the EEG-recording, mice were transferred to the testing room for acclimatization. Each mouse was weighed to calculate the exact treatment doses. For the control group, mice were treated with 200 µL of sterile injection water i.p., while drug-treated mice were injected with meclofenamate i.p. (Sigma-Aldrich, M4531-1G, dose: 20 mg/kg dissolved in sterile injection water). The treatment dose was determined based on our previous publication.^17^

### Induction of seizures with i.p. kainic acid injection

For seizure induction, mice were treated with i.p. KA (Sigma-Aldrich, 420318, dose: 10 mg/kg dissolved in sterile injection water, pH was not adjusted) 15 min after either vehicle or meclofenamate treatment.

### Video-EEG monitoring

Right after KA injection, mice were placed into a 25 cm × 25 cm transparent cage for video-EEG monitoring. To record the EEG waves of the hippocampus, a preamplifier was inserted into the six-pin connector *via* a multichannel commutator (Moflon Technology LTD, MC190), allowing the mice to freely explore and move within the cage. EEG signal was acquired at 1 kHz and band pass filtered at 1.6–2000Hz (Supertech BioAmp, Ad Instruments PowerLab). The video-EEG monitoring was performed for 90 min. We recorded EEG waves with LabChart software (Ad Instruments) and captured simultaneously behavioural activities/seizures with a webcam (Alcor AWC1080). Videos were scored based on the Racine’s scale, seizures were considered behavioural if Grade 3 or higher seizures were observed (forelimb clonus without rearing). At the end of the session, i.p. diazepam injection (dose: 5 mg/kg) was delivered. After the diazepam injection, animals were placed back to their home cages. All mice in our study experienced SE after KA injection and all of them survived for the next 3 weeks.

### Slice preparation

*In vitro* patch clamp recordings were performed in acute horizontal brain slices taken from naïve male C57BL/6N mice as it was described earlier.^11^ Since TRPM4 is more abundantly expressed in ventral MCs (see results), we used ventral slices for patch clamp studies.

### *In vitro* electrophysiological recordings

Patch clamp electrophysiological recordings were carried out as it was described earlier.^11^ When it is indicated, modified ACSF was used with 0 mM MgCl_2_ and 5 mM KCl. 10 μM meclofenamate (Sigma-Aldrich, 211281) 100 μM 4-Aminopyridine (Sigma-Aldrich 275875) or 1 μM Gabazin (Tocris 1262) was applied into the bath solution, when indicated.

### RNAscope *in situ* hybridization in mouse and human hippocampus

Four representative middle and ventral hippocampal sections of naïve C57BL/6 mice were used for RNAscope ISH. Two human hippocampal tissue blocks were coronally oriented, and the sections illustrated by Supplementary Fig. 7 were subjected to RNAscope ISH.

The RNAscope staining was optimized for PFA fixed tissue^21^ RNAscope experiments were performed based on the supplier instruction (ACD, Hayward, CA, USA) to visualize *Trpm4* (ACD; Cat. No.:534621) and *Cart* (ACD; Cat. No.: 432001-C3). After the RNAscope procedure, mouse samples were treated with the special AT-rich sequence-binding protein 1 (SATB1) primary antibody (mouse 1:250, SantaCruz, sc-376096) overnight. After washing, the slides were incubated with secondary antibody (donkey 1:500, Jackson ImmunoResearch Labs 715-585-150) for 3 h at room temperature in dark and then counterstained with DAPI (ACD), air-dried and cover-slipped with glycerol-PBS (1:1). RNAscope ISH was combined with immunofluorescence for CART to visualize the border of the TRPM4 positive neurons. After the RNAscope procedure, slides were treated with polyclonal rabbit anti-CART antibody (Phoenix H-003-62 (55–102)) diluted to 1:10.000, for 12 h at 24°C. After 2 min × 15 min washes, Alexa 488-conjugated donkey anti-rabbit antibody (Jackson Immunoresearch Europe Ltd., Cambridgeshire, United Kingdom; Cat. No: 711-545-152, diluted to 1:500) was used for 3 h. Sections were counterstained with DAPI (ACD) and covered with ProLong Gold Antifade (Thermo Fisher Scientific, Waltham, MA, USA) mounting medium*. Trpm4* mRNA signal appeared as well distinguishable fluorescent dots around the cell nucleus (stained with SATB1). The number of dots per cell was manually counted and averaged in 10 cells per section. In total, 10 middle sections and 10 ventral sections, from 3 mice were used. Mouse and human 3-plex positive (ACD; Cat. No: 320881) control probes specific to *Polr2a* mRNA (fluorescein), *Ppib* mRNA (Cy3), *Ubc* mRNA (cyanine 5, Cy5) and 3-plex negative (ACD; Cat. No: 320871) control probes to bacterial *dabP* mRNA were tested on mouse and human hippocampus. The 3-plex positive control probes produced clear signals in the human and mouse DG, while the negative control probes did not give any detectable fluorescence in the samples (Supplementary Fig. 1).

### Human hippocampus samples

Control subjects: SKO29R (right) HC3—perfusion-fixed and TBSKO1 HC—immersion-fixed (*n* = 2) studied in this project had no diagnosed neurological or psychiatric disorders or brain trauma, died from any not-brain-related cause and displayed no signs of brain neuropathologies (Supplementary Table 1).

Perfusion control: after the brain removal, perfusion *via* cannula inserted into the internal carotid and vertebral arteries was performed within a time window of 2 to 5 h post-mortem. Perfusion was performed with 1.5 L of 0.33% heparin containing physiological saline for 30 min, then with 4–5 L of a Zamboni fixative solution containing 4% paraformaldehyde and 0.2% picric acid in phosphate buffer (pH 7.4) over a duration of 1.5–2 h. After perfusion, 0.5–1 cm thick blocks were cut from the cortical regions of the brain. Regions were identified according to Brodmann division^22^ and post-fixed in the Zamboni solution overnight^23^

Immersion control: the brain removal was performed within 2 to 5 h *post-mortem*. The hippocampus were identified according to the Allen human brain atlas.^24^ After that tissue samples were immediately cut into 0.5–1 cm thick blocks and immersed into a Zamboni fixative containing 4% paraformaldehyde, and 0.2% picric acid in 0.1 M PB (the same fixative solution used for the brain perfusion). Fixative was changed hourly to a fresh solution during constant agitation for 6 h, and the blocks were then post-fixed overnight, in the same fixative^23^ Tissue samples of the hippocampal region were microdissected. The hippocampus were identified according to the Allen human brain atlas^24^ and post-fixed in the Zamboni solution overnight.^25^ The brain samples were sectioned for 30 μm thickness using a Leica VT1000S vibratome (Leica Biosystems, Wetzlar, Germany); then, the sections were collected and stored in sterile PBS, containing sodium azide (0.01%) at 4°C. For long-term storage at −20°C, they were transferred into anti-freeze solution.

The studies on perfused human brain samples received ethical approval from the Regional and Institutional Committee of Science and Research Ethics of the Scientific Council of Health (Hungary) (ETT TUKEB 15032/2019/EKU) and was conducted in adherence to the principles of the Declaration of Helsinki.

### Immunohistochemistry and confocal imaging

Two days following KA injection, animals were deeply anaesthetized and transcardially perfused with ice-cold saline followed by 4% paraformaldehyde in 0.1 M PBS (pH = 7.4). Brains were removed and postfixed in 4% paraformaldehyde solution. For immunohistochemistry, the right side of the brains was sliced with a vibratome (Leica, VS1000 s) into 40 μm thick sections in the horizontal plane. To determine MC loss after SE, six sections/mouse (three from central, three from ventral part) were collected and stained with SATB1 antibody. First, slices were washed 2 × in 0.1 M PBS and incubated with SATB1 antibodies (1:250, SantaCruz, sc-376096) overnight at 4 ^o^C. On the following day, slices were washed 2 × in 0.1 M PBS and incubated with a fluorescent dye (Alexa 594) conjugated donkey secondary antibodies raised against the host species of the primary antibody (1:500, Jackson ImmunoResearch Labs, 715-585-150) for 2 h at room temperature. Then, slices were washed 2 × in 0.1 M PBS, put on a glass slide and covered with ProLong Gold antifade reagent (Invitrogen, P36934).

To determine the cell loss after SE, confocal pictures were taken using a Nikon Eclipse Ti2-E confocal microscope (10 × and 20 × objectives), and all SATB1 positive cells in the hilus were counted using ImageJ software, finally average number of central and ventral MCs were calculated for each mouse. The analysis of the experiments was blind to the treatment of the animals.

### Statistical analysis

For spike analysis, we first defined the standard deviation (SD) for each recording from a spike free ‘baseline’ period at the beginning of the recordings using LabChart software. Spikes were regarded as fast events (> 10 Hz) with amplitude higher than 7 SDs of the given recording (Supplementary Fig. 2). Seizure was considered as train of spikes with a frequency of at least 0.5 Hz and a duration of at least 5 s. When it is not specified, the reported spike numbers are total spikes during both ictal and inter-ictal periods. Seizures with no behavioural signs were called electrographic seizure. Seizures with clear behavioural signs (Racine scale 3 or higher) were considered as behavioural seizure. Seizures were considered SE if appeared in both recording electrodes and persisted ≥ 5 min.^9^ The analysis of the EEG experiments was blind to the phenotype and the drug application. Spectral power analysis was performed on a 30 min recording period following the first seizure in each mice using LabChart (Ad Instruments) software. Data are represented as mean ± SD. The normality of samples was tested with Shapiro–Wilk test. Normally distributed samples were compared with two sample or paired sample Student’s *t*-test; non-normally distributed data were compared with Mann–Whitney test or Wilcoxon Signed rank test. ANOVA test with Tukey’s *post-hoc* test for normally distributed data were used for three or more group comparison by OriginPro 2016. Statistics are summarized in Supplementary Table 2.

## Results

### Meclofenamate treatment delays behavioural seizures after kainic acid injection

**Figure 1:**
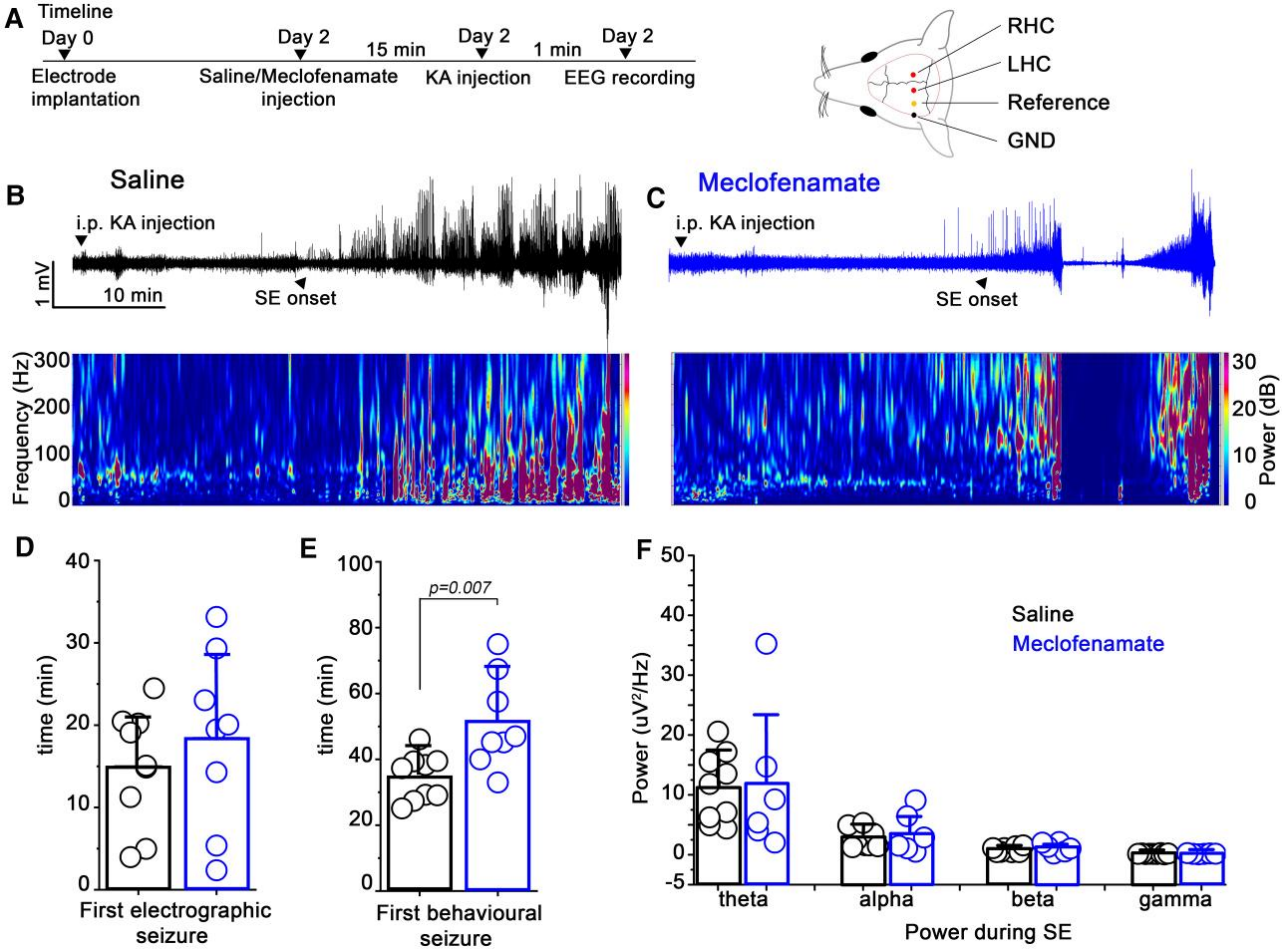
**Behavioural seizures are delayed after meclofenamate treatment.** (**A**) *Left,* Timeline of the experiments. Mice were implanted with EEG electrodes, 2 days later, they underwent saline- or meclofenamate-treatment and 15 min later kainic acid (KA) injection. Seizures were detected for 90 min. *Right*, Cartoon showing the position of the electrodes. (**B and C**) Representative EEG recordings showing the onset of status epilepticus (SE) (upper panel) and corresponding spectrograms (lower panel) in vehicle (black) and meclofenamate (blue) treated WT mice after KA injection. Statistics showing the (**D**) first electrographic seizure, the (**E**) first behavioural seizure and (**F**) power densities after seizure onset (30 min) (theta-*P* = 0.91, alpha-*P* = 0.59, beta-*P* = 0.32, gamma-*P* = 0.89). *N* = 9 for saline and 8 for meclofenamate, two sample *t*-test. Data are presented as mean ± SD. Each data points represent individual animals. GND, ground; LHC, left hippocampus; RHC, right hippocampus; SE, status epilepticus.

To evaluate the effect of meclofenamate on neuronal excitability during seizures, first electrodes were inserted into the left and right dorsal hippocampus. Two days later, mice received either an i.p. injection of meclofenamate or vehicle (control group) and 15 min later SE was induced with i.p. KA injection (Fig. 1A) a widely used model for experimental SE.^26,27^ Immediately after the KA injection, mice were connected to a video-EEG recording system to monitor seizures (Fig. 1B and C). These experiments revealed that the latency period prior to the first electrographic seizure (for definition of seizure see methods) was not different in the meclofenamate-injected compared to the vehicle-injected mice (saline = 14.92 ± 7.06 min, *n* = 9; meclofenamate = 18.38 ± 10.73 min, *n* = 8; *P* = 0.44, two sample *t*-test; Fig. 1D); however, the first behavioural seizure occurred significantly later (saline = 34.59 ± 7.09 min, *n* = 9; meclofenamate = 51.28 ± 14.22 min, *n* = 8; *P* = 0.007, two sample *t*-test) in meclofenamate-injected compared to vehicle-injected mice (Fig. 1E). After that, we analyzed spectral power during the first 30 min of SE focusing on the major frequency bands: theta (4–8 Hz), alpha (9–14 Hz), beta (15–30 Hz) and gamma (31–100 Hz) but found no significant changes in neither of the frequency bands (Fig. 1F and Supplementary Fig. 3).

### Meclofenamate reduces spiking activity after KA injection

**Figure 2:**
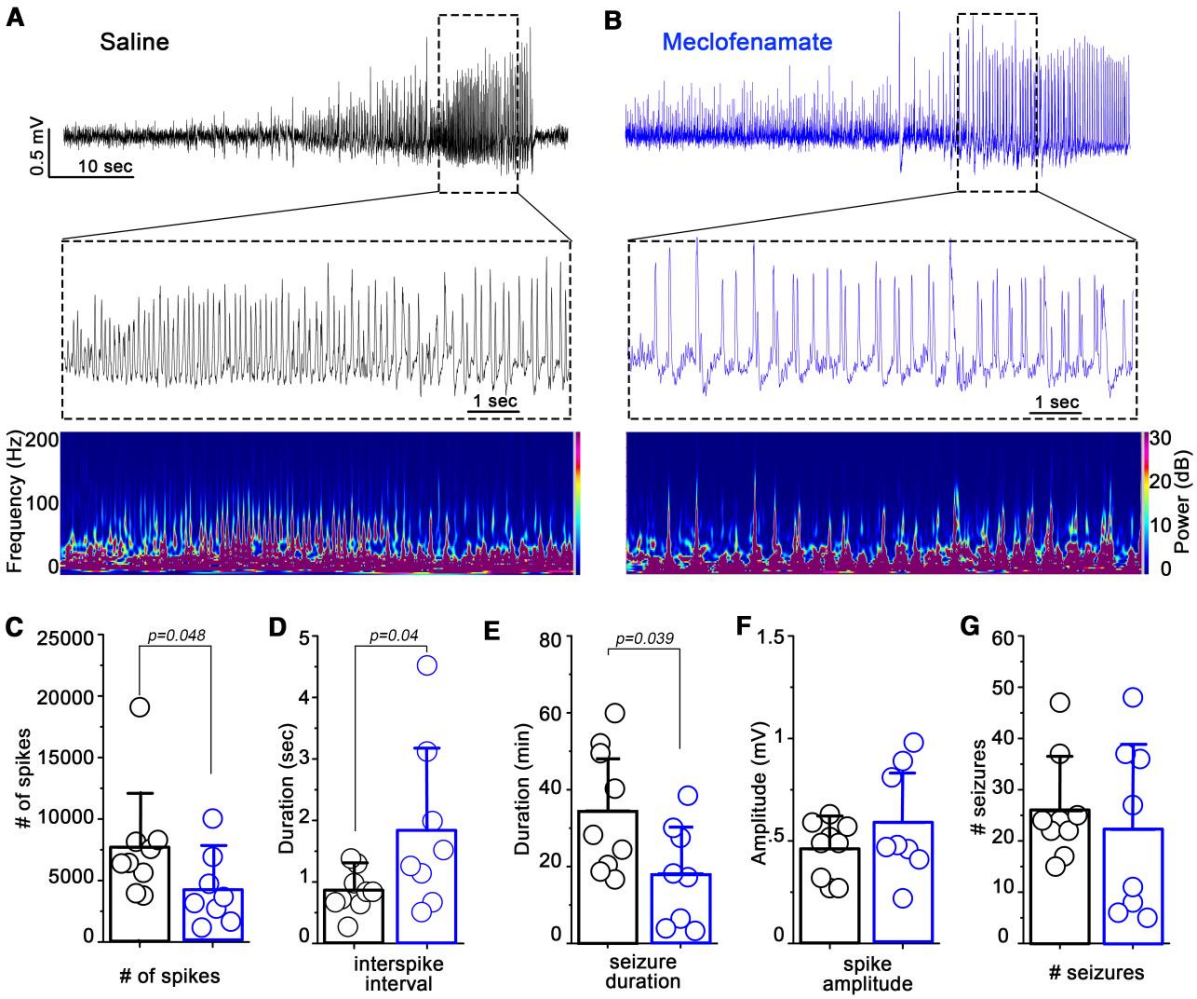
**Meclofenamate reduces spiking activity after KA injection.** (**A and B**) Representative EEG recordings of a behavioural seizure (*upper*) magnified view (*middle panel*) and corresponding spectrograms (*lower*) in saline (black) and meclofenamate (blue) treated WT mice after KA injection. Statistics showing the (**C**) number of spikes, the (**D**) interspike interval, the (**E**) total time spent with seizure, the (**F**) spike amplitudes and the (**G**) total number of seizures during the 90 min recording period after KA injection. *N* = 9 for saline and eight for meclofenamate, Mann–Whitney test (**C**); two sample *t*-test (**D–G**). Data are presented as mean ± SD. Each data points represent individual animals.

To further understand the effect of meclofenamate on KA induced seizures, we analyzed the spike transients including spike number, frequency and amplitude (Fig. 2A and B) during the whole recording period (90 min). Notably, meclofenamate-treatment significantly reduced the number of spikes (ictal and inter-ictal) (saline = 7699.88 ± 4573.22, *n* = 9; meclofenamate = 4259.62 ± 2950.32, *n* = 8; *P* = 0.048, Mann–Whitney test) and therefore increased the interspike interval (saline = 0.84 ± 0.34 s, *n* = 9; meclofenamate = 1.84 ± 1.35 s, *n* = 8; *P* = 0.05, two sample *t*-test) compared to vehicle treatment (Fig. 2C and D). Furthermore, total time spent with seizure during the 90 min recording period was also significantly shorter upon meclofenamate treatment (saline = 34.46 ± 16.27 min, *n* = 9; meclofenamate = 18.18 ± 13.10 min, *n* = 8; *P* = 0.039, two sample *t*-test; Fig. 2E). Of note, no difference was present between meclofenamate or vehicle treatment concerning the amplitude of the individual spikes (saline = 0.46 ± 0.13 mV, *n* = 9; meclofenamate = 0.59 ± 0.26 mV, *n* = 8; *P* = 0.22, two sample *t*-test; Fig. 2F) and the number of seizures (saline = 25.88 ± 10.03, *n* = 9; meclofenamate = 22.25 ± 16.83, *P* = 0.59, *n* = 8; two sample *t*-test; Fig. 2G). Next, we defined whether meclofenamate-treatment also decreased the occurrence and severity of chronic seizures. Six saline and seven meclofenamate-treated mice were used for 24 h EEG recordings 3–4 weeks after SE. In these experiments four out of six saline-injected while one out of seven meclofenamate-injected mice experienced spontaneous seizures (Supplementary Fig. 4). Notably, spike numbers and seizure numbers during the 24 h recording session (ictal and inter-ictal spikes) were significantly lower in the meclofenamate treated compared to saline treated group (saline = 1183.33 ± 1827.61, (ictal:531, inter-ictal:652.3) *n* = 6; meclofenamate = 501.7 ± 1198.83, (ictal:18.6, inter-ictal:483) *n* = 7; *P* = 0.026, Mann–Whitney test and saline = 2.83 ± 2.99, *n* = 6; meclofenamate = 0.14 ± 0.37, *n* = 7; *P* = 0.041, Mann–Whitney test, respectively). Although there was a tendency for decreased seizure duration as well (saline = 82.5 ± 143.96 s, *n* = 6; meclofenamate = 4.71 ± 12.47 s, *n* = 7; *P* = 0.061, Mann–Whitney test), these parameters were not significantly different.

### Meclofenamate has no anticonvulsive effect on Trpm4^−/−^ mice

**Figure 3:**
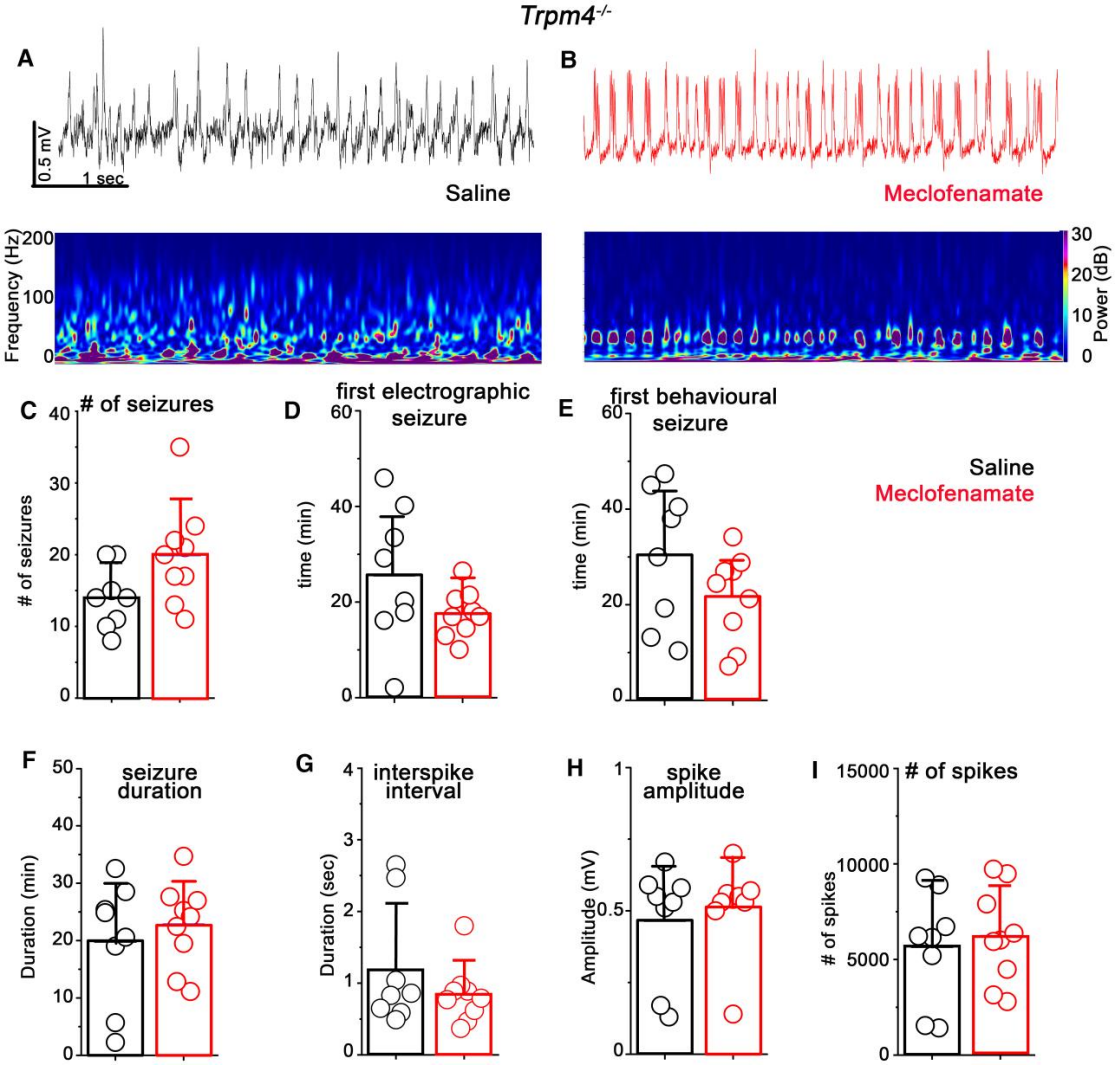
**Meclofenamate has no anticonvulsive effect on *Trpm4^−/−^* mice.** (**A and B**) Representative EEG recordings (*upper*) and corresponding spectrograms (*lower*) in vehicle- (black) and meclofenamate-treated (red) mice after kainic acid (KA) injection in *Trpm4^−/−^*mice. Statistics showing the (**C**) total number of seizures (*P* = 0.054, t = −2.08), the (**D**) first electrographic seizure (*P* = 0.13, t = 1.5), the (**E**) first behavioural seizure (*P* = 0.15, t = 1.5) the (**F**) total seizure duration (*P* = 0.52, t = −0.64), the (**G**) interspike interval (*P* = 0.28, t = 1.11), the (**H**) spike amplitudes (*P* = 0.59, t = −0.54), and the (**I**) number of spikes during the 90 min recording period after KA injection (*P* = 0.69, t = −0.4). *N* = 8 for *Trpm4^−/−^*_saline_ and nine for *Trpm4^−/−^*_meclofenamate_, two-sample *t*-test. Data are presented as mean ± SD. Each data points represent individual animals.

To test whether the anticonvulsant effect of meclofenamate is TRPM4 specific, we repeated the above detailed experiments on *Trpm4^−/−^* mice (Fig. 3A and B). Interestingly, meclofenamate-treatment had no significant effect on neither of the analyzed parameters including the number of seizures (saline = 14 ± 4.37, *n* = 8; meclofenamate = 20 ± 7.01, *n* = 9; *P* = 0.054, two sample *t*-test), the first electrographic (saline = 25.64 ± 14.3 min, *n* = 8; meclofenamate = 17.57 ± 4.89 min, *n* = 9; *P* = 0.13, two sample *t*-test) and behavioural seizures (saline = 30.46 ± 14.54 min, *n* = 8; meclofenamate = 21.72 ± 9.13 min, *n* = 9; *P* = 0.15, two sample *t*-test), the total time spent with seizure (saline = 19.87 ± 10.71 min, *n* = 8; meclofenamate = 22.74 ± 7.38 min, *n* = 9; *P* = 0.52, two sample *t*-test), the interspike interval (saline = 1.19 ± 0.85 s, *n* = 8; meclofenamate = 0.84 ± 0.41 s, *n* = 9; *P* = 0.28, two sample *t*-test), the spike amplitude (saline = 0.46 ± 0.2 mV, *n* = 8; meclofenamate = 0.51 ± 0.15 mV, *n* = 9; *P* = 0.59, two sample *t*-test) and the number of spikes (saline = 5677.25 ± 2932.79, *n* = 6; meclofenamate = 6208.22 ± 2500.77, *n* = 6; *P* = 0.69, two sample *t*-test; Fig. 3C–I). Interestingly, some of the seizure parameters in the vehicle-treated *Trpm4^−/−^* animals were similar to those of meclofenamatetreated WT mice including seizure duration and number of spikes indicating that genetic ablation or pharmacological blocking of TRPM4 results in similar phenotype in terms of seizure susceptibility.

### Ventral MCs are protected after KA injection in meclofenamate treated WT mice

**Figure 4:**
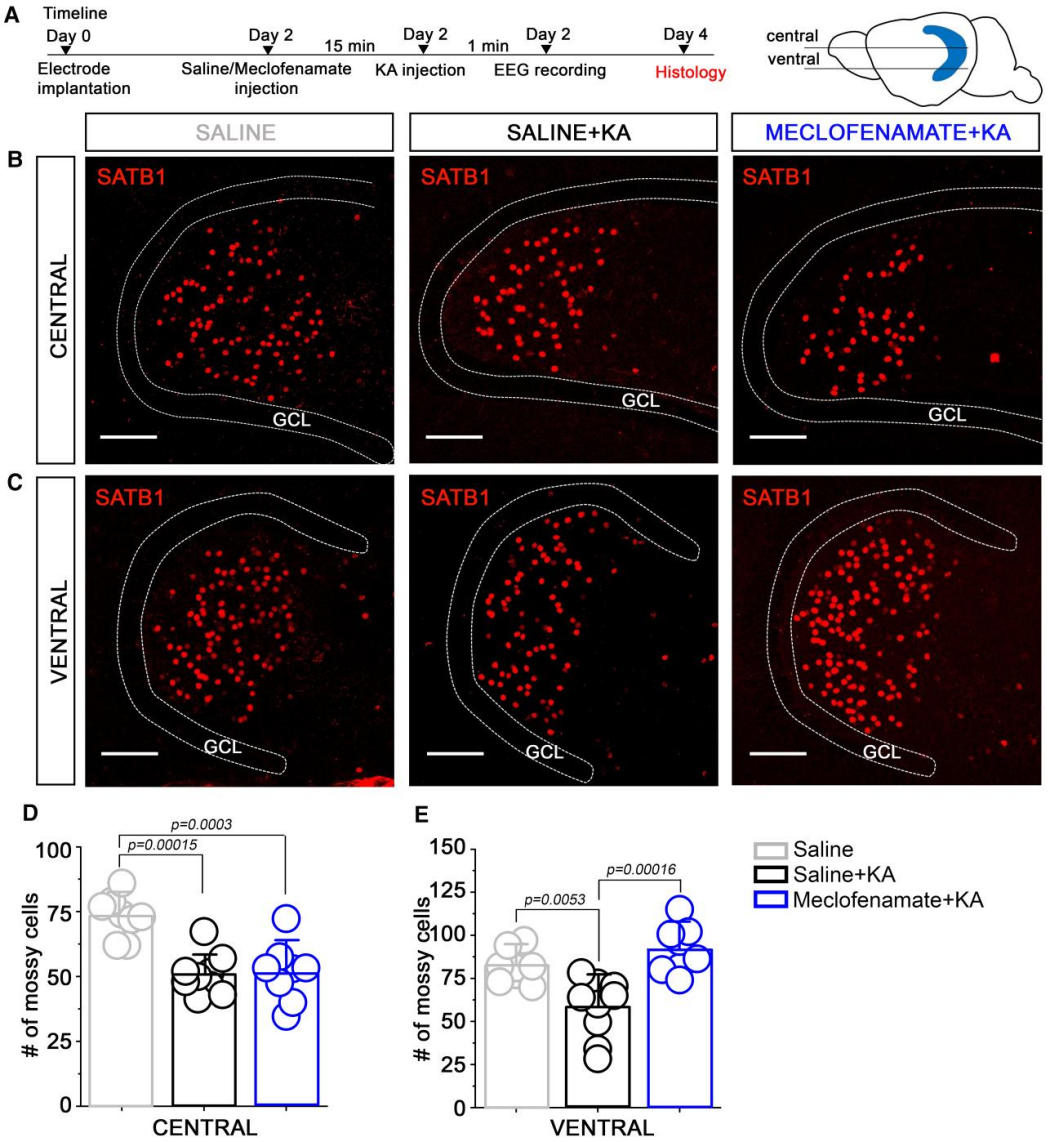
**Ventral mossy cells are protected after KA injection in meclofenamate-treated WT mice.** (**A**) left: Timeline of the experiments: 2 days after SE induction (detailed in Figs 2 and 3) mice were sacrificed for histological studies. *Right*: cartoon showing the positions of the histological slices. Representative confocal images of SATB1 immunopositive neurons in the central (**B**) and ventral hippocampal (**C**) layer from saline, saline + KA and meclofenamate + KA-treated WT mice. Statistics showing the number of mossy cells in saline, saline + KA and meclofenamate + KA-treated mice from the central (**D**) and ventral (**E**) hippocampus. *n* = 8 for saline_central_, nine for saline + KA_central_ and eight for meclofenamate + KA_central_, eight for saline_ventral,_ nine for saline + KA_ventral_ and eight for meclofenamate + KA_ventral_, One-way ANOVA, Tukey’s *post-hoc* test. Data points represent average MC numbers from individual animals (from each mouse 3 section from central and 3 from ventral region were used to calculate an average). Data are presented as mean ± SD. Scale bar is 100 µm. GCL, granule cell layer.

To examine the effect of meclofenamate-treatment in MC vulnerability, 2 days after the SE experiments (i.p. KA injection) mice were sacrificed and their whole brain was removed (Fig. 4A). Horizontal brain slices were immunostained with the previously identified MC specific marker SATB1.^11^ From each mouse, six sections were collected: three from the middle and three from the ventral part of the hippocampus, the number of MCs *per* section was determined and averaged for each mouse (Fig. 4B and C). These experiments revealed that meclofenamate-treatment did not affect the surviving rate of MCs in the central hippocampus (saline = 50.7 ± 7.82, *n* = 9; meclofenamate = 51.47 ± 11.39, *n* = 8; *P* = 0.98, one-way ANOVA, Tukey’s *post-hoc*). However, we found a significantly higher number of surviving MCs in the ventral hippocampus upon meclofenamate-treatment (saline = 58 ± 17.28, *n* = 19; meclofenamate = 91.93 ± 13.81, *n* = 17; *P* = 1.62 × 10^−4^, one-way ANOVA, Tukey’s *post-hoc*; Fig. 4D and E). Of note, MC number in saline-injected control mice in central and ventral hippocampus were 73.12 ± 8.1, *n* = 8 and 82 ± 9.41, *n* = 8, respectively indicating that KA injection reduced MC number in both regions, but meclofenamate-treatment was able to reduce MC loss in the ventral hippocampus.

### *Trpm4* is more abundantly expressed in ventral MCs

**Figure 5:**
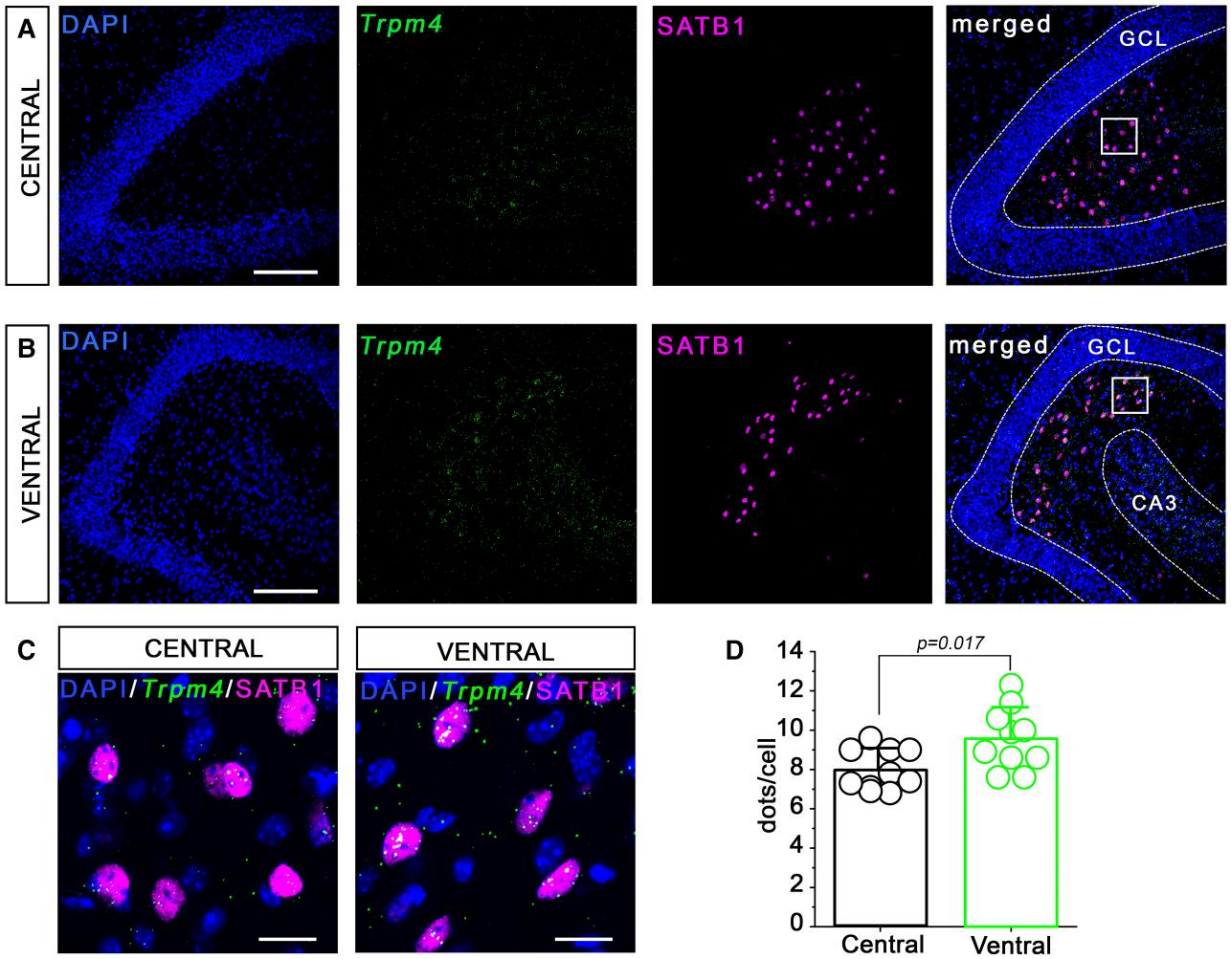
**Higher *Trpm4* expression in ventral compared to central mossy cells (MCs).** Representative confocal images (**A–C**) of RNAscope labelling of *Trpm4* (green) and immunofluorescence staining of SATB1 (magenta) as a marker of mossy cells in central and ventral hippocampus. Cell nuclei are stained with DAPI (blue). Note, that SATB1 stains exclusively the nucleus of neurons; therefore, *Trpm4* signal in the proximity of SATB1 staining is likely in the cytoplasm of mossy cells. Significantly more RNAscope transcripts were found in the ventral compared to central MCs (**D**). Data points represent hippocampal slices (RNAscope puncta averaged from 10 cells/slice). *n* = 10 dorsal slices and 10 ventral slices, from three mice were used. Data are presented as mean ± SD, two sample *t*-test. CA3: cornu Ammonis area 3. Scale bar 100 µm (**A** and **B**) and 5 µm (**C**). GCL, granular cell layer.

Clearly, the better survival rate of ventral compared to central MCs upon meclofenamate treatment following KA injection raised a vital question: is there a difference between these cell populations? It has been previously shown that dorsal and ventral MCs are not entirely homogeneous cell populations regarding their axonal projections.^28,29^ However, the most obvious explanation to our finding could be that dorsal and ventral MCs might not be homogenous in terms of *Trpm4* expression too. To address this possibility, we performed RNAscope ISH to detect *Trpm4* mRNA transcripts given the controversy in the literature concerning the immune-histochemical alternatives.^30^  *Trpm4* RNAscope and SATB1 immunostaining outcomes reinforced our previous finding that SATB1 positive MCs also express *Trpm4* (Fig. 5A–C).^11^ Interestingly, quantification of the RNAscope signal showed that ventral MCs contain significantly more *Trpm4* transcripts compared to central MCs (central = 7.99 ± 1.04, *n* = 10; ventral = 9.55 ± 1.57, *n* = 10; *P* = 0.017, two sample *t*-test; Fig. 5D).

### Meclofenamate influences intrinsic electrophysiological properties of MCs

**Figure 6:**
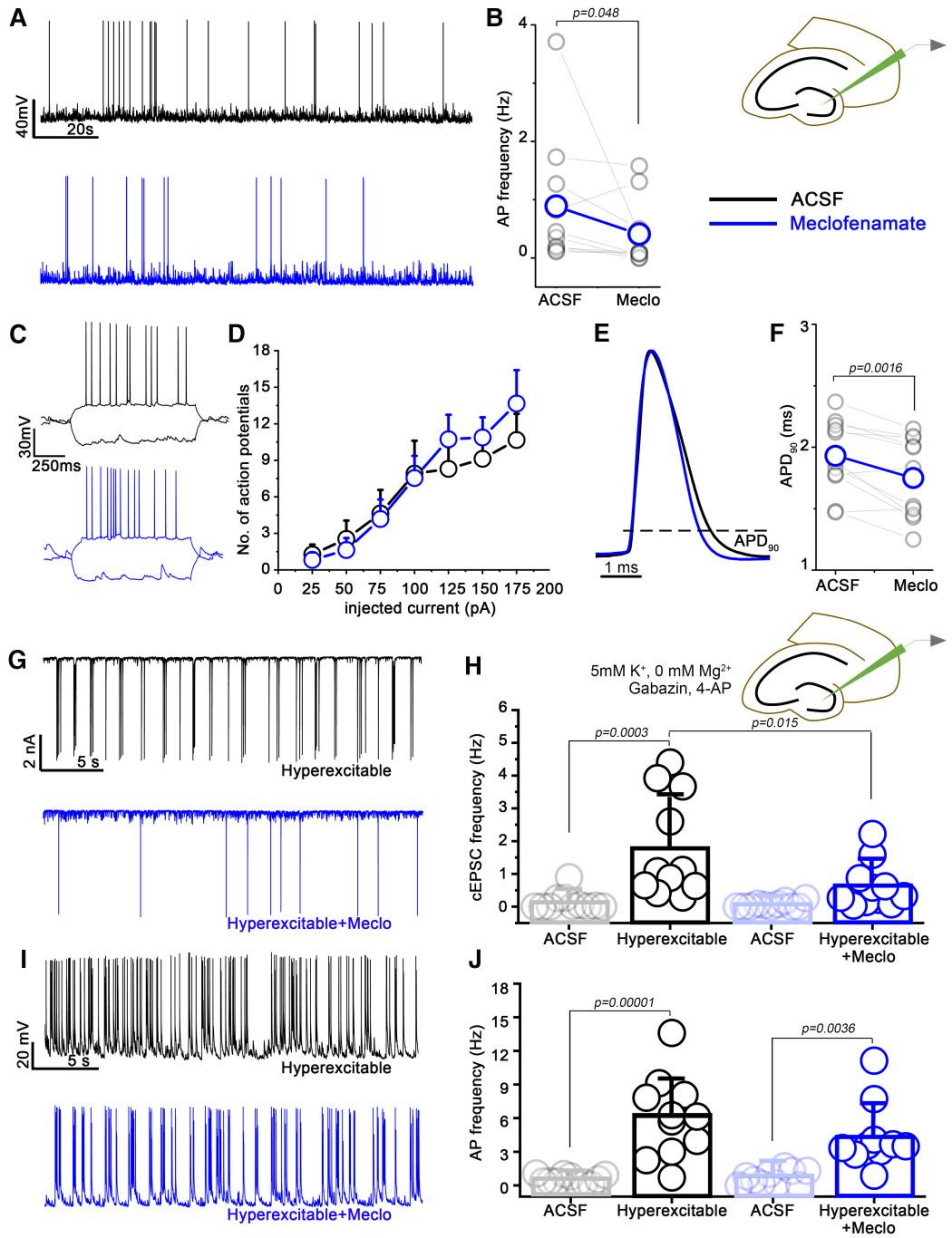
**Meclofenamate modifies electrophysiological properties of mossy cells (MCs).** (**A**) Representative spontaneous action potentials (APs) from MCs in control conditions (black) and upon meclofenamate-treatment (blue). (**B**) Statistics of AP frequency in control conditions (*n* = 10, black) and upon meclofenamate-treatment (*n* = 10, blue), Wilcoxon Signed Ranks Test. (**C**) Representative voltage traces from MCs in control conditions (black) and upon meclofenamate-treatment (blue). (**D**) Current versus firing rate relationship in control conditions (*n* = 11, black) and upon meclofenamate-treatment (*n* = 11, blue). Two sample T-test (p/t values: 25 pA = 0.66/0.44, 50 A = 0.61/0.5, 75 pA = 0.85/0.18, 100 pA = 0.91/0.11, 125 pA = 0.38/−0.89, 150 pA = 0.5/−0.68, 175 pA = 0.4/−0.86). (**E**) Overlay of APs from MCs in control conditions (black) and upon meclofenamate-treatment (blue). Amplitudes of the representative APs are normalized to each other for better visibility. (**F**) Statistics of AP duration at 90% of AP amplitude (APD_90_) from MCs in control conditions (*n* = 11, black) and upon meclofenamate-treatment (*n* = 11, blue) paired sample *t*-test. (**G**) Representative voltage clamp traces from MCs during hyperexcitable conditions in control (black) and upon meclofenamate-pretreatment (blue). (**H**) Statistics showing the frequency of cEPSCs in control conditions (*n* = 11, black) and upon meclofenamate treatment (*n* = 11, blue), one-way ANOVA. (**I**) Representative current clamp traces from MCs during hyperexcitable conditions in control (black) and upon meclofenamate-pretreatment (blue). (**J**) Statistics showing the frequency of spontaneous APs in control conditions (*n* = 11, black) and upon meclofenamat- treatment (*n* = 10, blue) one-way ANOVA. Data are presented as mean ± SD. Each data point represents recording from individual MCs, one MC was measured per each animal.

To test the direct effect of meclofenamate on MCs, we performed whole-cell patch clamp recordings in horizontal brain slices containing the hippocampal formation. In certain cases, biocytin-filled cells were stained with SATB1 for *post-hoc* identification, as described earlier.^11^ We and others showed previously that MCs are intrinsically active and TRPM4 might modulate this activity.^11,31^ Therefore, we explored whether meclofenamate has any effect on the intrinsic activity of MCs. Using the current clamp configuration, we showed that the frequency of the spontaneous APs was significantly lower upon meclofenamate treatment (ACSF = 0.89 ± 1.12 Hz, *n* = 10; meclofenamate = 0.41 ± 0.57 Hz, *n* = 10; *P* = 0.048, Wilcoxon signed ranks test; Fig. 6A and B). Next, we asked whether meclofenamate has any effect on the intrinsic excitability of MCs including changes in the threshold for AP. Thus, MCs were injected with increasing steps of current pulses to depolarize the cells and trigger AP firing in control conditions (ACSF) and in the presence of meclofenamate (Fig. 6C and D) while the cells’ resting membrane potential were held at ∼ −65 mV. These experiments revealed that meclofenamate did not change the threshold to elicit APs. We and others demonstrated previously that TRPM4 as a Ca^2+^ activated cationic current can prolong the repolarization of the AP.^32,33^ Interestingly, meclofenamate treatment shortened the AP, measured at 90% of the repolarization (ACSF = 1.93 ± 0.28 ms, *n* = 11; meclofenamate = 1.75 ± 0.31 ms, *n* = 11; *P* = 0.0016, paired sample *t*-test) indicating a possible blockade of TRPM4 (Fig. 6E and F). Of note, the above-mentioned parameters were not significantly changed in *Trpm4^−/−^* MCs upon meclofenamate treatment (Supplementary Fig. 5). Basic electrophysiological properties including resting membrane potential, input resistance, membrane time constant and cell capacitance were not significantly different upon meclofenamate treatment (Supplementary Fig. 6). To further evaluate the role of meclofenamate on MC excitability during epileptic conditions, we stimulated seizure-like events *in vitro* by depolarization (zero Mg^2+^ and high extracellular K^+^), gamma-aminobutyric acid receptor blockade (Gabazine) and K^+^-channel blockade (4-AP). Spontaneous activity of MCs was recorded in both current and voltage clamp mode first in standard recording conditions and then during pharmacologically induced hyperexcitability. The *in vitro* pharmacological stimulation resulted in very large amplitude spontaneous bursts of compound excitatory postsynaptic currents in voltage clamp mode as described earlier^34^ and in high frequency AP firing in current clamp mode (Fig. 6G and I). Interestingly, the compound excitatory postsynaptic currents on MCs were significantly reduced if meclofenamate was administered (1 min) prior the drug application (Hyperexcitable = 1.78 ± 1.55 Hz, *n* = 11; Hyperexcitable + meclo = 0.62 ± 0.69 Hz, *n* = 11; *P* = 0.015, one-way ANOVA, Tukey’s *post-hoc*; Fig. 6H). In comparison, the frequency of APs after drug application were not statistically different (Hyperexcitable = 6.05 ± 3.61 Hz, *n* = 11; Hyperexcitable + meclo = 4.36 ± 2.91 Hz, *n* = 10; *P* = 0.36, one-way ANOVA, Tukey’s *post-hoc*; Fig. 6J) although the same tendency was present.

### TRPM4 is expressed in human MCs

**Figure 7:**
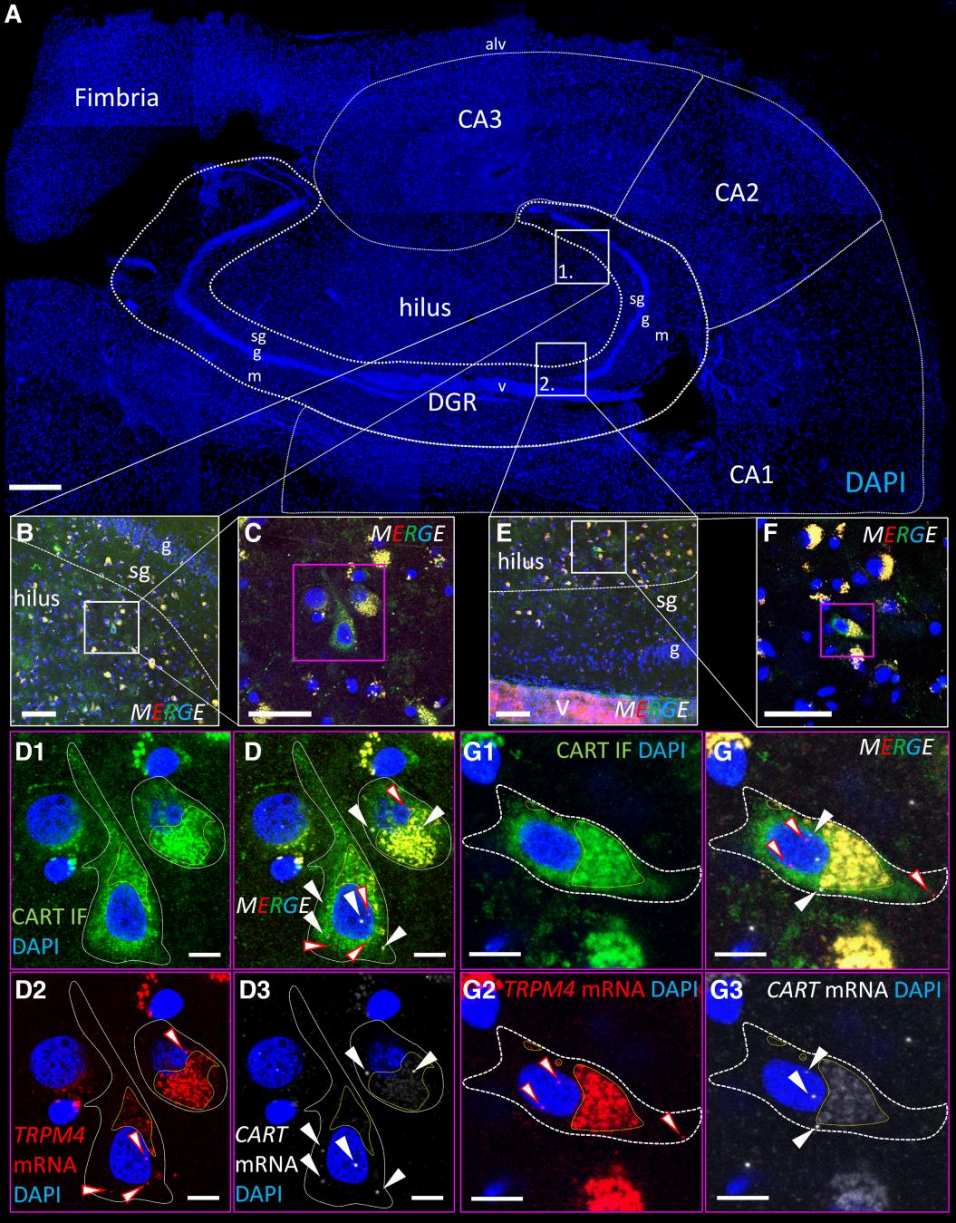
**Human hippocampal mossy cells express *TRPM4* mRNA.** (**A**) Shows a montage of stitched confocal images that show the coronal section of the human hippocampus. The borders of the main hippocampal areas were indicated: cornu Ammonis (CA)1, CA2, CA3 and the dentate gyrus (DGR) with its molecular (m), granular (g) and subgranular (sg) layer. The fimbria, the alveus (alv) and a longitudinally cut thicker blood vessel (v) in the DGR are also recognizable. The boxed area no. 1 is shown in higher magnification in **B** upon immunofluorescence labelling (IF) for cocain- and amphetamine-regulated transcript peptide (CART, green) combined with RNAscope in situ hybridization (ISH) for *TRPM4* (red) and *CART* (white) mRNAs. (**C**) Indicates ideally cut mossy cells in the boxed area of **B**. The magenta boxed area is indicated with high magnification in **D**. The individual channels illustrating CART IF (green, D_1_), *TRPM4* mRNA ISH (red, D_2_), and *CART* mRNA (white, D_3_) were merged with the channel of 4′,6-diamidino-2-phenylindole (DAPI, blue) nuclear counterstaining. The boxed area no. 2 is indicated in **E**. A mossy cell that was ideal for imaging is further magnified in **F**. (**G**) Shows the same mossy cell in high magnification that is visible in the magenta box in **F**. (**G_1_**) Illustrates the CART IF, G_2_ the *TRPM4* mRNA, while G_3_ the *CART* mRNA. In the high magnification images of **D** and **G**, the borders of the cells were indicated by white dotted lines, based on the green CART IF that is a marker of human mossy cells (see our reference Seress *et al.*^35^). The yellow dotted line borders cytoplasmic areas, where lipofuscin accumulation is observable, that causes some autofluorescence in all channels resulting in a yellow shade in the merge images. The RNAscope ISH signal puncta that indicate individual mRNA transcripts were highlighted by white arrowheads for *CART* mRNA and white arrowheads with red borders for *TRPM4* transcripts. Note, that we identified a number of signal dots inside the karyoplasma that does not contain lipofuscin, besides some cytoplasmic signal puncta. The merge (**D**, **G**) high magnification images illustrate that these signal dots do not correspond to lipofuscin as they are not yellow. Bars: 1 mm in **A**, 100 μm in **B** and **E**, 25 μm in **C** and **F**, 5 μm in **D** and **G**.

To map the expression of *TRPM4* in human MCs, we performed RNAscope *in situ* hybridization to detect even very low level of mRNA on hippocampus samples from *post-mortem* human brain (Supplementary Fig. 7). To identify MCs within the hilar region, we performed a duplex RNAscope staining as well as immunostaining using a probe against the cocaine- and amphetamine-regulated transcript peptide (*CART*) as a molecular marker.^35^ These experiments revealed that *TRPM4* mRNA is present in the *CART-*positive human MCs from both samples (Fig. 7 and Supplementary Fig. 8).

## Discussion

In this study, we report that the nonsteroidal anti-inflammatory drug meclofenamate effectively curtailed spiking activity induced by KA. Specifically, we demonstrated that *in vivo* application of meclofenamate reduced the duration of epileptic seizures, the number of spikes and the onset of behavioural seizures. Moreover, MC loss often seen after SE was reduced in the ventral region of the hippocampus upon meclofenamate treatment. These effects of meclofenamate are most likely TRPM4 specific, since application of meclofenamate in *Trpm4^−/−^* mice had no protective effect in the KA model. Furthermore, using patch clamp recordings, we showed that meclofenamate alters spontaneous activity and AP dynamics of MCs. Finally, we demonstrated that human MCs also express *TRPM4.*

In our previous work, both the morphological and functional presence of *Trpm4* in hilar MCs were reported for the first time along with the finding that TRPM4 as a Ca^2+^-activated cation channel regulates MCs’ intrinsic electrophysiological properties.^11^ Our findings indicated that the intracellular Ca^2+^ increase during synaptic events activates TRPM4 and amplifies the original input. However, this amplifier function turns into a drawback during pro-epileptic insults and can worsen the cellular damage caused by excitotoxicity. Our results therefore highlighted that TRPM4 might be a promising drug target in seizure management. Interestingly, it has been shown recently that TRPM4 and NMDA receptor coupling can cause excitotoxicity in neurons.^36^ However, the most often used TRPM4 blockers are partially selective^13,14^ and have unfavourable pharmacokinetics *in vivo.*^12^ Recently, we identified meclofenamate as a potent TRPM4 antagonist and proved *in vivo* that it has a so far unrecognized anti-arrhythmic effect *via* TRPM4 blockade in the heart.^17^

Meclofenamate is a nonsteroidal anti-inflammatory drug, and it is a potent inhibitor of the enzyme cyclooxygenase, thereby inhibiting prostaglandin production. It is a Food and Drug Administration approved drug; however, it is mostly replaced by diclofenac nowadays. Interestingly, previous reports had already suggested that meclofenamate might have anticonvulsive effect by blocking gap junctions or activating KCNQ2/3 potassium channels; however, the exact mechanism remained elusive.^37,38^ Recently, it has been shown that meclofenamate can inhibit CLC-2 chloride channels as well regulating cellular excitability.^39^On the other hand, to our best knowledge, no data are available about the functional expression of neither KCNQ2/3 potassium channels nor CLC-2 chloride channels in MCs. Clearly, more experiments are needed to clarify the role of these channels in MCs if any. Recently, we identified that meclofenamate is a promising blocker of TRPM4, since unlike the other known TRPM4 inhibitors (9-phenantrol, flufenamic acid) meclofenamate has favourable water solubility and pharmacokinetics and its effect on other ion channels is limited.^17,37^

Using KA as a proconvulsant to induce SE and later the development of spontaneously recurring seizures is a frequently used epilepsy model in mouse.^27^ In our experiments, meclofenamate-treatment prior to the administration of KA remarkably reduced the severity of seizures. Of note, it reduced the time spent with seizures, the number of spikes and the onset of behavioural seizures. Additionally, spontaneous seizures and spike numbers were also less frequent in meclofenamate-treated mice weeks after SE, most likely because of the reduced initial epileptogenic insult. However, the first electrographic seizure was not delayed upon meclofenamate-treatment. What could be the target of meclofenamate to achieve such antiseizure effect? In our next set of experiments, we found that meclofenamate has no anticonvulsive effect at all using *Trpm4^−/−^* mice indicating that it most likely acts *via* TRPM4 inhibition. Considering our previous observations that TRPM4 acts as an amplifier on EPSPs, one can speculate that blocking TRPM4 can lead to decreased MC excitability and therefore can reduce spiking during seizures. Interestingly, it has been shown recently that flufenamaic acid, the non-specific blocker of TRPM4 can abolishes epileptiform activity in the entorhinal cortex.^40^

It has to be noted as well that SE also leads to brain inflammation which can effect epileptogenesis.^41^ Since meclofenamate is a NSAID one can assume that its anti-inflammatory effect plays a role in our observations rather than its blocking effect on TRPM4. However, the onset of inflammatory processes starts the earliest 2–3 h after SE while we observed clear anti-seizure effects during the first 90 min after KA injection.^42^ Furthermore, we did not see any effect of meclofenamate on *Trpm4^−/−^* mice suggesting that the reported outcome of meclofenamate-treatment is more likely lies on the blockade of TRPM4 rather than its anti-inflammatory effect or its off-target effect *via* other ion channels.

Hippocampal cell loss is a common characteristic of SE both in rodents and human patients.^43,44^ Interestingly, it has been shown earlier that glibenclamide—a non-specific TRPM4 antagonist—can improve cell loss in status epilepticus.^45^ Among other cell types, hilar MCs are the most vulnerable to epileptic insults.^46^ Here, we demonstrated that meclofenamate treatment reduced MC loss after KA injection. Intriguingly, the reduced loss was present in the ventral but not in the middle part of the hippocampus. It has been previously shown that MCs might not homogenous across the dorsoventral axis of the hippocampus. Two independent studies found that dorsal and ventral MCs differ significantly in their axonal projections and therefore may have different functional roles.^28,29^ Our results extend these findings about inhomogeneity of MCs with a higher expression of TRPM4 in the ventral part. One can speculate that ventral MCs might be more vulnerable to KA-induced seizures because of the higher TRPM4 expression and higher excitability. Interestingly, early studies already suggested that there is a subpopulation of MCs with lower threshold for perforant path activation.^47^ Clearly, further studies are needed to clarify the physiological and pathophysiological role of different MC subpopulations.

We and others showed previously that TRPM4 as a Ca^2+^-activated cation channel contributes to neuronal excitability, AP dynamics and can support pacemaker activity.^11,48^ Blocking TRPM4 with 9-phenantrol can decrease spontaneous activity of MCs and abolish subthreshold membrane potential oscillations on pacemaker neurons.^11,48^ Here, we showed that meclofenamate did not change the threshold for AP firing in MCs. Nevertheless, it decreased their spontaneous AP firing indicating that most likely the amplitudes of the excitatory inputs were reduced by meclofenamate. Furthermore, meclofenamate shortened the AP duration indicating the blockade of a Ca^2+^ activated depolarizing current at the late phase of the AP. Of note, the electrophysiological changes caused by meclofenamate application are very similar to those found in *Trpm4^−/−^* MCs.^11^ In our voltage clamp recordings, we occasionally detected large amplitude compound EPSCs, which were heavily increased in frequency upon hyperexcitable conditions (low Mg^2+^ high extracellular K^+^, GABAR and K^+^ channel blockade). These compound excitatory postsynaptic currents are proposed to arise from highly synchronized firing of the recurrent cornu Ammonis (CA) 3 network, and they were previously recorded in epileptic rats.^34,49,50^ Interestingly, meclofenamate decreased the frequency of these compound excitatory postsynaptic currents further supporting our hypothesis that TRPM4 has a role in amplifying synaptic input onto MCs.

Finally, using human *post-mortem* hippocampus, we provide here the first direct evidence that *TRPM4* is expressed in human MCs as well, emphasizing the translational aspects of our study. Of note, we must also mention that TRPM4 is expressed in various other cell types besides neurons^10^; therefore, systemic administration of a TRPM4 blocker must be handled with special caution because of the possible side effects.

Taken together, we report here a so far unknown antiseizure effect of the nonsteroidal anti-inflammatory drug meclofenamate *via* blocking TRPM4 in hilar MCs. Previously we already showed that deletion of TRPM4 can protect MCs during acute seizures.^11^ However, as a limitation of our previous work, it must be noted that global deletion of a protein may lead to uncertain compensatory changes, which might affect our experimental results. The main finding of the current study is that we were able to show that blocking TRPM4 acutely in a WT mice results in a very similar antiseizure effect as it was shown in *Trpm4^−/−^* mice. These findings are specifically important since MC cell loss is one of the major hallmark of TLE^7^ thus, protecting MCs during insults often leading to temporal lobe epilepsy such as SE might be antiepileptogenic.^9^ Of note, meclofenamate is already an Food and Drug Administration approved drug thus it could be an ideal candidate for drug repositioning.^51^ Finally, our results further support that besides cardiac pathologies, TRPM4 is a promising drug target in seizure management, demonstrating the urgent need for more potent and more selective TRPM4 antagonists.

## Supplementary Material

fcaf229_Supplementary_Data

## Data Availability

The data that support the findings of this study are available from the corresponding author upon reasonable request.

## References

[1] Amaral DG, Scharfman HE, Lavenex P. The dentate gyrus: Fundamental neuroanatomical organization (dentate gyrus for dummies). Prog Brain Res. 2007;163:3–22. 17765709 10.1016/S0079-6123(07)63001-5 PMC2492885

[2] Kesner RP . An analysis of dentate gyrus function (an update). Behav Brain Res. 2018;354:84–91. 28756212 10.1016/j.bbr.2017.07.033

[3] Scharfman HE . The enigmatic mossy cell of the dentate gyrus. Nat Rev Neurosci. 2016;17(9):562–575. 27466143 10.1038/nrn.2016.87 PMC5369357

[4] Buckmaster PS. Mossy fiber sprouting in the dentate gyrus. In: Noebels JL, Avoli M, Rogawski MA, Olsen RW, Delgado-Escueta AV, eds. 2012. Oxford University Press, USA; 2012:479–499. 22787642

[5] Soriano E, Frotscher M. Mossy cells of the rat fascia dentata are glutamate-immunoreactive. Hippocampus. 1994;4(1):65–69. 7914798 10.1002/hipo.450040108

[6] Ribak CE, Seress L, Amaral DG. The development, ultrastructure and synaptic connections of the mossy cells of the dentate gyrus. J Neurocytol. 1985;14(5):835–857. 2419523 10.1007/BF01170832

[7] Kecskés A, Czéh B, Kecskés M. Mossy cells of the dentate gyrus: Drivers or inhibitors of epileptic seizures? Biochim Biophys acta Mol cell Res 2022;1869(9):119279. 35526721 10.1016/j.bbamcr.2022.119279

[8] Bui AD, Nguyen TM, Limouse C, et al Dentate gyrus mossy cells control spontaneous convulsive seizures and spatial memory. Science. 2018;359(6377):787–790. 29449490 10.1126/science.aan4074 PMC6040648

[9] Botterill JJ, Lu YL, LaFrancois JJ, et al An excitatory and epileptogenic effect of dentate gyrus mossy cells in a mouse model of epilepsy. Cell Rep. 2019;29(9):2875–2889.e6. 31775052 10.1016/j.celrep.2019.10.100 PMC6905501

[10] Mathar I, Jacobs G, Kecskes M, Menigoz A, Philippaert K, Vennekens R. TRPM4. Handb Exp Pharmacol. 2014;222:461–487. 24756717 10.1007/978-3-642-54215-2_18

[11] Mundrucz L, Kecskés A, Henn-Mike N, et al TRPM4 regulates hilar mossy cell loss in temporal lobe epilepsy. BMC Biol. 2023;21(1):96. 37101159 10.1186/s12915-023-01604-3 PMC10134545

[12] Guinamard R, Hof T, Del Negro CA. The TRPM4 channel inhibitor 9-phenanthrol. Br J Pharmacol. 2014;171(7):1600–1613. 24433510 10.1111/bph.12582 PMC3966741

[13] Burris SK, Wang Q, Bulley S, Neeb ZP, Jaggar JH. 9-Phenanthrol inhibits recombinant and arterial myocyte TMEM16A channels. Br J Pharmacol. 2015;172(10):2459–2468. 25573456 10.1111/bph.13077 PMC4409899

[14] Veress R, Baranyai D, Hegyi B, et al Transient receptor potential melastatin 4 channel inhibitor 9-phenanthrol inhibits K(+) but not Ca(2+) currents in canine ventricular myocytes. Can J Physiol Pharmacol. 2018;96(10):1022–1029. 29806985 10.1139/cjpp-2018-0049

[15] Guinamard R, Simard C, Del Negro C. Flufenamic acid as an ion channel modulator. Pharmacol Ther. 2013;138(2):272–284. 23356979 10.1016/j.pharmthera.2013.01.012 PMC4116821

[16] Ozhathil LC, Delalande C, Bianchi B, et al Identification of potent and selective small molecule inhibitors of the cation channel TRPM4. Br J Pharmacol. 2018;175(12):2504–2519. 29579323 10.1111/bph.14220 PMC6002741

[17] Vandewiele F, Pironet A, Jacobs G, et al TRPM4 inhibition by meclofenamate suppresses Ca2+-dependent triggered arrhythmias. Eur Heart J. 2022;43(40):4195–4207. 35822895 10.1093/eurheartj/ehac354

[18] Conroy MC, Randinitis EJ, Turner JL. Pharmacology, pharmacokinetics, and therapeutic use of meclofenamate sodium. Clin J Pain. 1991;7(Suppl 1):S44–S48. 1810520

[19] Scharfman HE, MacLusky NJ. Sex differences in the neurobiology of epilepsy: A preclinical perspective. Neurobiol Dis. 2014;72(Pt B):180–192. 25058745 10.1016/j.nbd.2014.07.004 PMC4252793

[20] Vennekens R, Olausson J, Meissner M, et al Increased IgE-dependent mast cell activation and anaphylactic responses in mice lacking the calcium-activated nonselective cation channel TRPM4. Nat Immunol. 2007;8(3):312–320. 17293867 10.1038/ni1441

[21] Nemes B, Bölcskei K, Kecskés A, et al Human somatostatin SST(4) receptor transgenic mice: Construction and brain expression pattern characterization. Int J Mol Sci. 2021;22(7):3758. 33916620 10.3390/ijms22073758 PMC8038480

[22] Brodmann K . Vergleichende lokalisationslehre der grosshirnrinde in ihren prinzipien dargestellt auf grund des zellenbaues. Leipzig. 1909.

[23] Maglóczky Z, Halász P, Vajda J, Czirják S, Freund TF. Loss of calbindin-D28 K immunoreactivity from dentate granule cells in human temporal lobe epilepsy. Neuroscience. 1997;76(2):377–385. 9015323 10.1016/s0306-4522(96)00440-x

[24] Ding SL, Royall JJ, Sunkin SM, et al Comprehensive cellular-resolution atlas of the adult human brain. J Comp Neurol. 2016;524(16):3127–3481. 27418273 10.1002/cne.24080 PMC5054943

[25] Szekeres-Paraczky C, Szocsics P, Erőss L, Fabó D, Mód L, Maglóczky Z. Reorganization of parvalbumin immunopositive perisomatic innervation of principal cells in focal cortical dysplasia type IIB in human epileptic patients. Int J Mol Sci. 2022;23(9):4746. 35563137 10.3390/ijms23094746 PMC9100614

[26] Umpierre AD, Bennett IV, Nebeker LD, et al Repeated low-dose kainate administration in C57BL/6J mice produces temporal lobe epilepsy pathology but infrequent spontaneous seizures. Exp Neurol. 2016;279:116–126. 26896834 10.1016/j.expneurol.2016.02.014 PMC5382800

[27] Lévesque M, Avoli M. The kainic acid model of temporal lobe epilepsy. Neurosci Biobehav Rev. 2013;37(10 Pt 2):2887–2899. 24184743 10.1016/j.neubiorev.2013.10.011 PMC4878897

[28] Houser CR, Peng Z, Wei X, Huang CS, Mody I. Mossy cells in the dorsal and ventral dentate gyrus differ in their patterns of axonal projections. J Neurosci. 2021;41(5):991–1004. 33268544 10.1523/JNEUROSCI.2455-20.2020 PMC7880284

[29] Botterill JJ, Gerencer KJ, Vinod KY, Alcantara-Gonzalez D, Scharfman HE. Dorsal and ventral mossy cells differ in their axonal projections throughout the dentate gyrus of the mouse hippocampus. Hippocampus. 2021;31(5):522–539. 33600026 10.1002/hipo.23314 PMC8247909

[30] Fearey BC, Binkle L, Mensching D, et al A glibenclamide-sensitive TRPM4-mediated component of CA1 excitatory postsynaptic potentials appears in experimental autoimmune encephalomyelitis. Sci Rep. 2022;12(1):6000. 35397639 10.1038/s41598-022-09875-6 PMC8994783

[31] Scharfman HE . Electrophysiological evidence that dentate hilar mossy cells are excitatory and innervate both granule cells and interneurons. J Neurophysiol. 1995;74(1):179–194. 7472322 10.1152/jn.1995.74.1.179

[32] Mathar I, Kecskes M, Van der Mieren G, et al Increased β-adrenergic inotropy in ventricular myocardium from Trpm4-/- mice. Circ Res. 2014;114(2):283–294. 24226423 10.1161/CIRCRESAHA.114.302835

[33] Simard C, Hof T, Keddache Z, Launay P, Guinamard R. The TRPM4 non-selective cation channel contributes to the mammalian atrial action potential. J Mol Cell Cardiol. 2013;59:11–19. 23416167 10.1016/j.yjmcc.2013.01.019

[34] Hedrick TP, Nobis WP, Foote KM, Ishii T, Chetkovich DM, Swanson GT. Excitatory synaptic input to hilar mossy cells under basal and hyperexcitable conditions. eNeuro. 2017;4(6):1–14. 10.1523/ENEURO.0364-17.2017 PMC5714709 29214210

[35] Seress L, Abrahám H, Dóczi T, Lázár G, Kozicz T. Cocaine- and amphetamine-regulated transcript peptide (CART) is a selective marker of rat granule cells and of human mossy cells in the hippocampal dentate gyrus. Neuroscience. 2004;125(1):13–24. 15051141 10.1016/j.neuroscience.2003.12.035

[36] Yan J, Bengtson CP, Buchthal B, Hagenston AM, Bading H. Coupling of NMDA receptors and TRPM4 guides discovery of unconventional neuroprotectants. Science. 2020;370(6513):eaay3302. 33033186 10.1126/science.aay3302

[37] Peretz A, Degani N, Nachman R, et al Meclofenamic acid and diclofenac, novel templates of KCNQ2/Q3 potassium channel openers, depress cortical neuron activity and exhibit anticonvulsant properties. Mol Pharmacol. 2005;67(4):1053–1066. 15598972 10.1124/mol.104.007112

[38] Manjarrez-Marmolejo J, Franco-Pérez J. Gap junction blockers: An overview of their effects on induced seizures in animal models. Curr Neuropharmacol. 2016;14(7):759–771. 27262601 10.2174/1570159X14666160603115942 PMC5050393

[39] Koster AK, Reese AL, Kuryshev Y, et al Development and validation of a potent and specific inhibitor for the CLC-2 chloride channel. Proc Natl Acad Sci U S A. 2020;117(51):32711–32721. 33277431 10.1073/pnas.2009977117 PMC7768775

[40] Sinyak DS, Amakhin DV, Soboleva EB, Gryaznova MO, Zaitsev AV. Flufenamic acid abolishes epileptiform activity in the entorhinal cortex slices by reducing the temporal summation of glutamatergic responses. Biochem Biophys Res Commun. 2024;733:150666. 39244848 10.1016/j.bbrc.2024.150666

[41] Rana A, Musto AE. The role of inflammation in the development of epilepsy. J Neuroinflammation. 2018;15(1):144. 29764485 10.1186/s12974-018-1192-7 PMC5952578

[42] Tilelli CQ, Flôres LR, Cota VR, de Castro OW, Garcia-Cairasco N. Amygdaloid complex anatomopathological findings in animal models of status epilepticus. Epilepsy Behav. 2021;121(Pt B):106831. 31864944 10.1016/j.yebeh.2019.106831

[43] Mathern GW, Adelson PD, Cahan LD, Leite JP. Hippocampal neuron damage in human epilepsy: Meyer’s hypothesis revisited. Prog Brain Res. 2002;135:237–251. 12143344 10.1016/s0079-6123(02)35023-4

[44] Blümcke I, Suter B, Behle K, et al Loss of hilar mossy cells in Ammon’s horn sclerosis. Epilepsia. 2000;41(Suppl 6):S174–S180. 10999540 10.1111/j.1528-1157.2000.tb01577.x

[45] Lin Z, Huang H, Gu Y, et al Glibenclamide ameliorates cerebral edema and improves outcomes in a rat model of status epilepticus. Neuropharmacology. 2017;121:1–11. 28412320 10.1016/j.neuropharm.2017.04.016

[46] Goldberg EM, Coulter DA. Mechanisms of epileptogenesis: A convergence on neural circuit dysfunction. Nat Rev Neurosci. 2013;14(5):337–349. 23595016 10.1038/nrn3482 PMC3982383

[47] Scharfman HE . Dentate hilar cells with dendrites in the molecular layer have lower thresholds for synaptic activation by perforant path than granule cells. J Neurosci. 1991;11(6):1660–1673. 2045880 10.1523/JNEUROSCI.11-06-01660.1991 PMC6575404

[48] Li K, Abbott SBG, Shi Y, Eggan P, Gonye EC, Bayliss DA. TRPM4 mediates a subthreshold membrane potential oscillation in respiratory chemoreceptor neurons that drives pacemaker firing and breathing. Cell Rep. 2021;34(5):108714. 33535052 10.1016/j.celrep.2021.108714 PMC7888550

[49] Scharfman HE . Synchronization of area CA3 hippocampal pyramidal cells and non-granule cells of the dentate gyrus in bicuculline-treated rat hippocampal slices. Neuroscience. 1994;59(2):245–257. 8008190 10.1016/0306-4522(94)90593-2 PMC3286025

[50] Scharfman HE, Smith KL, Goodman JH, Sollas AL. Survival of dentate hilar mossy cells after pilocarpine-induced seizures and their synchronized burst discharges with area CA3 pyramidal cells. Neuroscience. 2001;104(3):741–759. 11440806 10.1016/s0306-4522(01)00132-4 PMC2518406

[51] Jourdan JP, Bureau R, Rochais C, Dallemagne P. Drug repositioning: A brief overview. J Pharm Pharmacol. 2020;72(9):1145–1151. 32301512 10.1111/jphp.13273 PMC7262062

