# Supplementary material for: The nonsteroidal anti-inflammatory drug meclofenamate mitigates kainic acid–induced seizures via TRPM4 inhibition
Source: Brain Commun. 2025 Jun 12;7(3):fcaf229. doi: 10.1093/braincomms/fcaf229 (PMC12199760; doi:10.1093/braincomms/fcaf229)
Supplement: fcaf229_Supplementary_Data [file fcaf229_supplementary_data.pdf]

The nonsteroidal anti-inflammatory drug meclofenamate mitigates kainic acid-induced seizures *via* TRPM4 inhibition

Erzsébet Kövesdi[1#], Laura Mundrucz[1#], Attila Gyéresi[1], Máté Deák[1], Balázs Gaszner[2], Andy Pironet[3], Cecília Szekeres-Paraczky[4,5], Zsófia Maglóczky[4], Péter Gombás[6], Rudi Vennekens[3], Viktória Kormos[7 ŧ] and Miklós Kecskés[1ŧ *]

[1] *Institute of Physiology, Medical School, University of Pécs, H-7624, Pécs, Hungary*

[2] *Department of Anatomy, Medical School and Research Group for Mood Disorders, Centre for Neuroscience, University of Pécs, H-7624, Pécs, Hungary*

[3] *Laboratory of Ion Channel Research, Biomedical Sciences Group, Department of Cellular and Molecular Medicine, VIB-KU Leuven Center for Brain & Disease Research, KU Leuven, 3000, Leuven, Belgium*

[4] *Human Brain Research Laboratory, HUN-REN Institute of Experimental Medicine, H-1083 Budapest, Hungary*

[5] *Szentágothai János Doctoral School of Neuroscience, Semmelweis University, 1085 Budapest, Hungary*

[6] *Department of Pathology, St. Borbála Hospital, H-2800 Tatabánya, Hungary*

[7] *Department of Pharmacology and Pharmacotherapy, Centre for Neuroscience, Medical School, University of Pécs, H-7624, Pécs, Hungary*

[#]Erzsébet Kövesdi and Laura Mundrucz contributed equally to this work

[ŧ]Viktória Kormos and Miklós Kecskés contributed equally to this work

[*]Corresponding author: kecskes.miklos@pte.hu

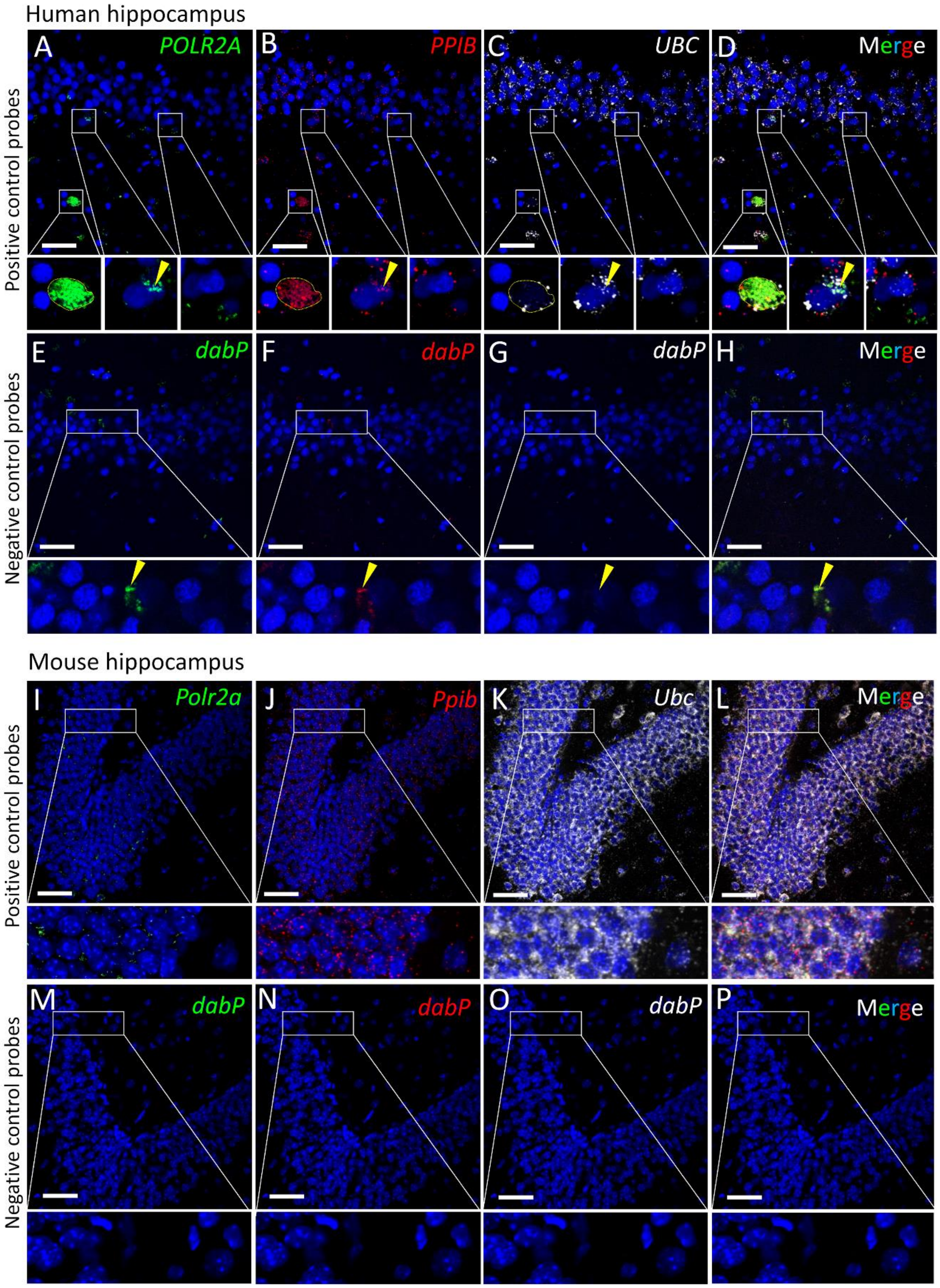
Human hippocampus
Positive control probes
Negative control probes
A
POLR2A
B
PPIB
C
UBC
D
Merge
E
dabP
F
dabP
G
dabP
H
Merge
Mouse hippocampus
Positive control probes
Negative control probes
I
Polr2a
J
Ppib
K
Ubc
L
Merge
M
dabP
N
dabP
O
dabP
P
Merge

Supplementary Figure 1. Sections were hybridized with triplex positive control probes for the human or mouse, and also with a triplex negative control probe. Positive control probes (A-D and I-L) provided clear fluorescent signal puncta in the low copy (DNA-directed RNA polymerase II subunit RPB1 mRNA, (green, *POLR2A* (human) or *Polr2a* (mouse)), and mid copy (peptidyl-prolyl cis-trans isomerase B mRNA (red, *PPIB* (human) *Ppib* (mouse)) channels. Confluent fluorescence was detected with the high copy (polyubiquitin-C mRNA, white, *UBC* (human) or *Ubc* (mouse)) positive control. No fluorescence was recognizable in any channels (*i.e.* green, red, white) when a negative control probe, designed to recognize bacterial dihydrodipicolinate reductase *(dabP)* mRNA, was applied. Nuclear counterstaining was performed by 4′,6-diamidino-2-phenylindole (blue). In the human sample (A-H), considerable lipofuscin accumulation caused autofluorescence. In panel A-D, a cell with stronger lipofuscin accumulation (yellow dotted line marked area in the left lower boxes in the inserts) is depicted. The middle boxed cell showed moderate lipofuscin (yellow arrowhead) accumulation. The third box indicates cells in the granular layer of the dentate gyrus which did not accumulate considerable amount of lipofuscin. Note that the autofluorescence of lipofuscin can be observed in all channels (yellow arrowheads). In contrast, the individual RNAscope signal dots are not detectable in all channels, but only in one. This can be observed in the merged images, especially in high magnification, where the specific signal puncta do not overlap. In the negative control (E-H), beyond some lipofuscin that is detected in all channels, no specific fluorescence signal is visible. In the mouse, the lipofuscin accumulation was negligible. No specific fluorescent signal was visible in any negative control RNAscope channels (I-L). Bars: 50µm.

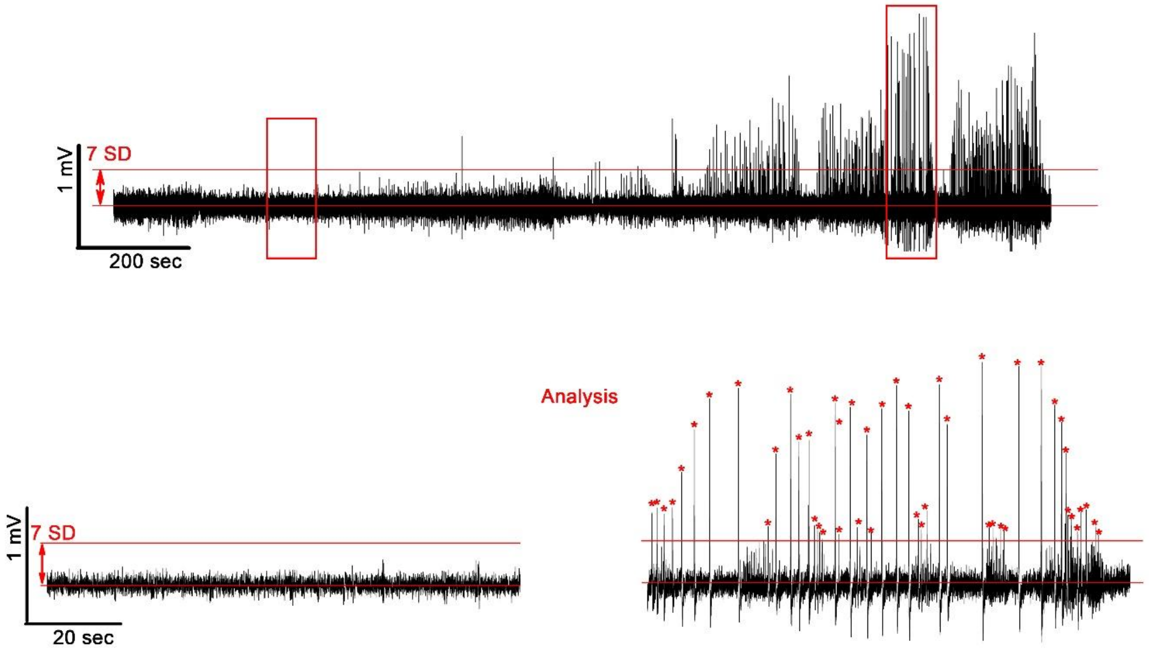

Supplementary Figure 2.

Representative recordings showing the onset of status epilepticus after kainic acid injection (upper panel). A threshold of 7 standard deviation (SD) of the baseline EEG amplitude was set as the criteria for spike detection (lower left). Spikes (red asterisk) that exceeded the ≥7 SD threshold criteria were analyzed.

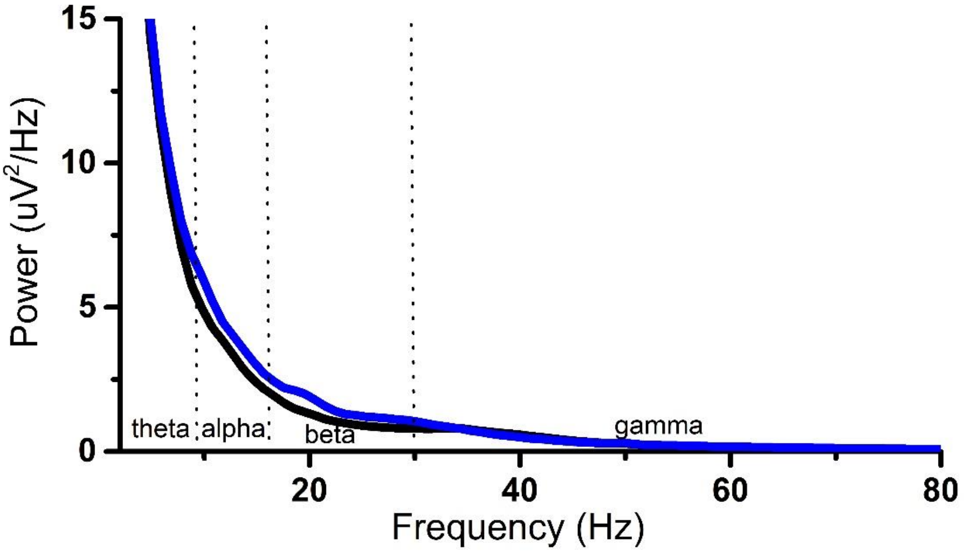

Supplementary Figure 3.

Power spectrum of control (black) and meclofenamate treated (blue) WT mice are not different during SE.

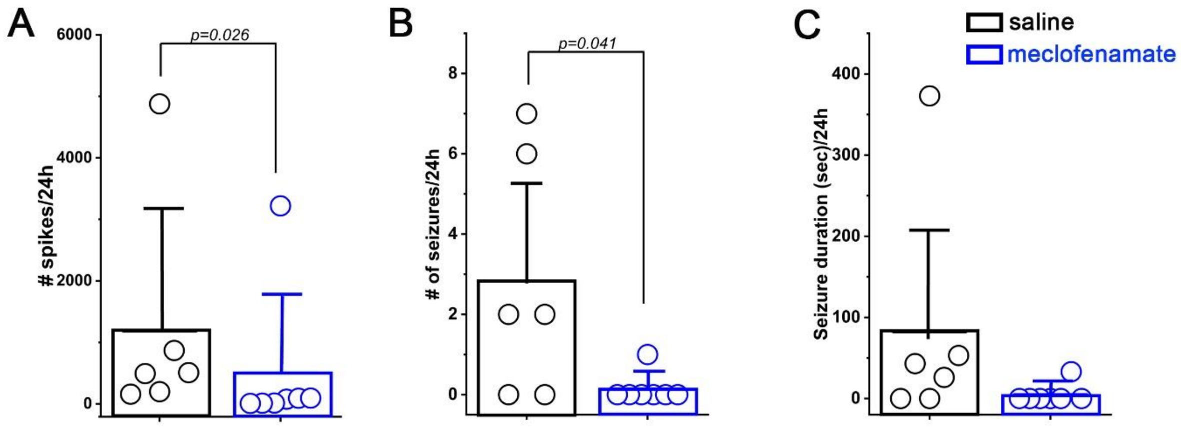

Supplementary Figure 4.

Statistics showing the (A) number of spikes the (B) total seizure numbers and the (C) total time spent with seizure during the 24 hours recording period 3-4 weeks after KA injection. n=6 for saline and 7 for meclofenamate. Data are presented as mean ± SD. Mann-Whitney test. Data points represents individual animals.

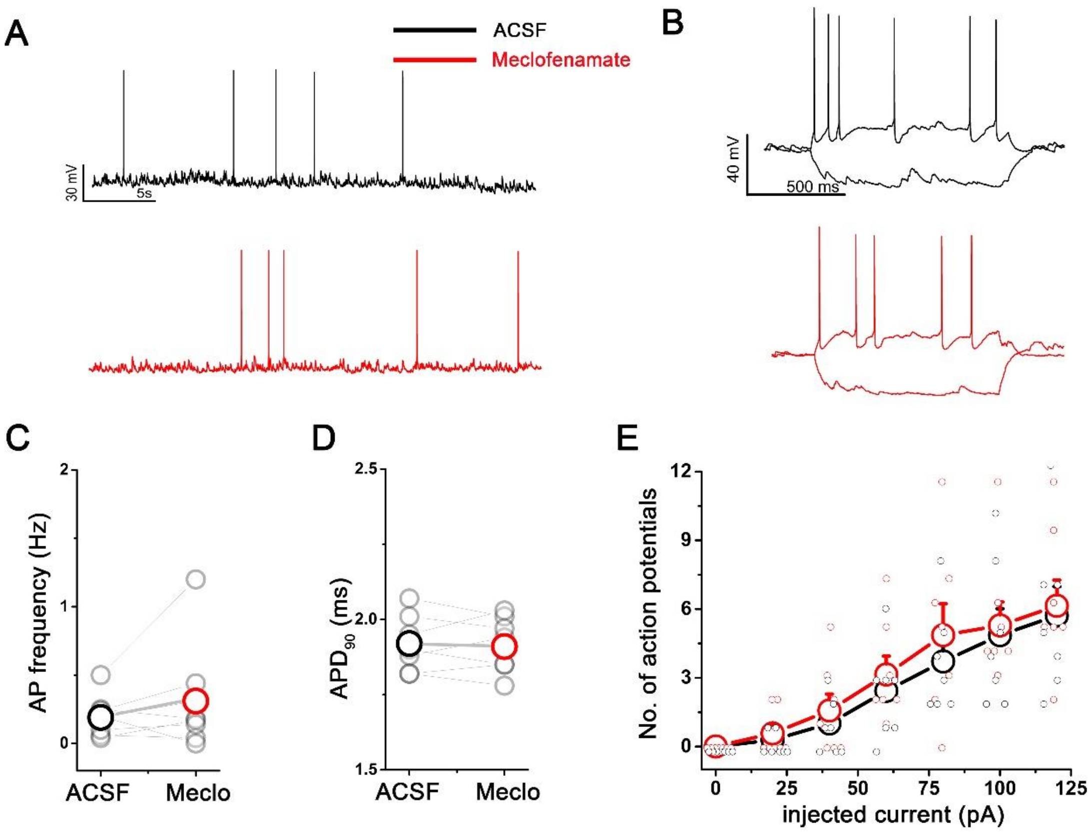

Supplementary Figure 5.

(A) Representative spontaneous action potentials (APs) from *Trpm4*$^{-/-}$ MCs in control conditions (black) and upon meclofenamate treatment (red). **(B)** Representative voltage traces from *Trpm4*$^{-/-}$ MCs in control conditions (black) and upon meclofenamate treatment (red). **(C)** Statistics of spontaneous AP frequency in control conditions (n=7) and upon meclofenamate treatment (n=7) in *Trpm4*$^{-/-}$ MCs (Wilcoxon Signed Rank Test p=0.32, Z=-0.98). **(D)** Statistics of AP duration at 90% of AP amplitude (APD_{90}) from *Trpm4*$^{-/-}$ MCs in control conditions (n=7) and upon meclofenamate treatment (n=7) (Paired Sample T-test p=0.4, t=-0.88). **(E)** Current versus firing rate relationship in control conditions (n=7, black) and upon meclofenamate treatment (n=7, red) in *Trpm4*$^{-/-}$ MCs Two sample T-test (p/t values: 20pA=0.5/0.44, 40pA=0.51/0.5, 60pA=0.52/0.18, 80pA=0.49/0.11, 100pA=0.78/-0.89, 120pA=0.8/-0.68). Data are presented as mean ± SD. Paired sample T-test. Data points represent individual cells.

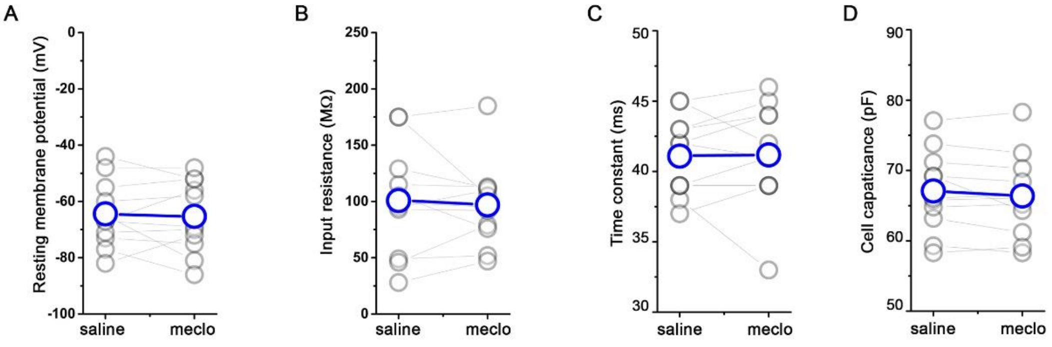

Supplementary Figure 6.

Statistics showing the resting membrane potential (p=0.71, t=0.37) (A), input resistance (p=0.64, t=0.46) (B), membrane time constant (p=0.89, t=-0.13) (C) and cell capacitance (p=0.19, t=1.37) (D) of saline and meclofenamate treated MCs. n=11 for saline and 11 for meclofenamate, Paired Student T-test. Data are presented as mean. Data points represent individual MCs, one MC was measured *per* each animal.

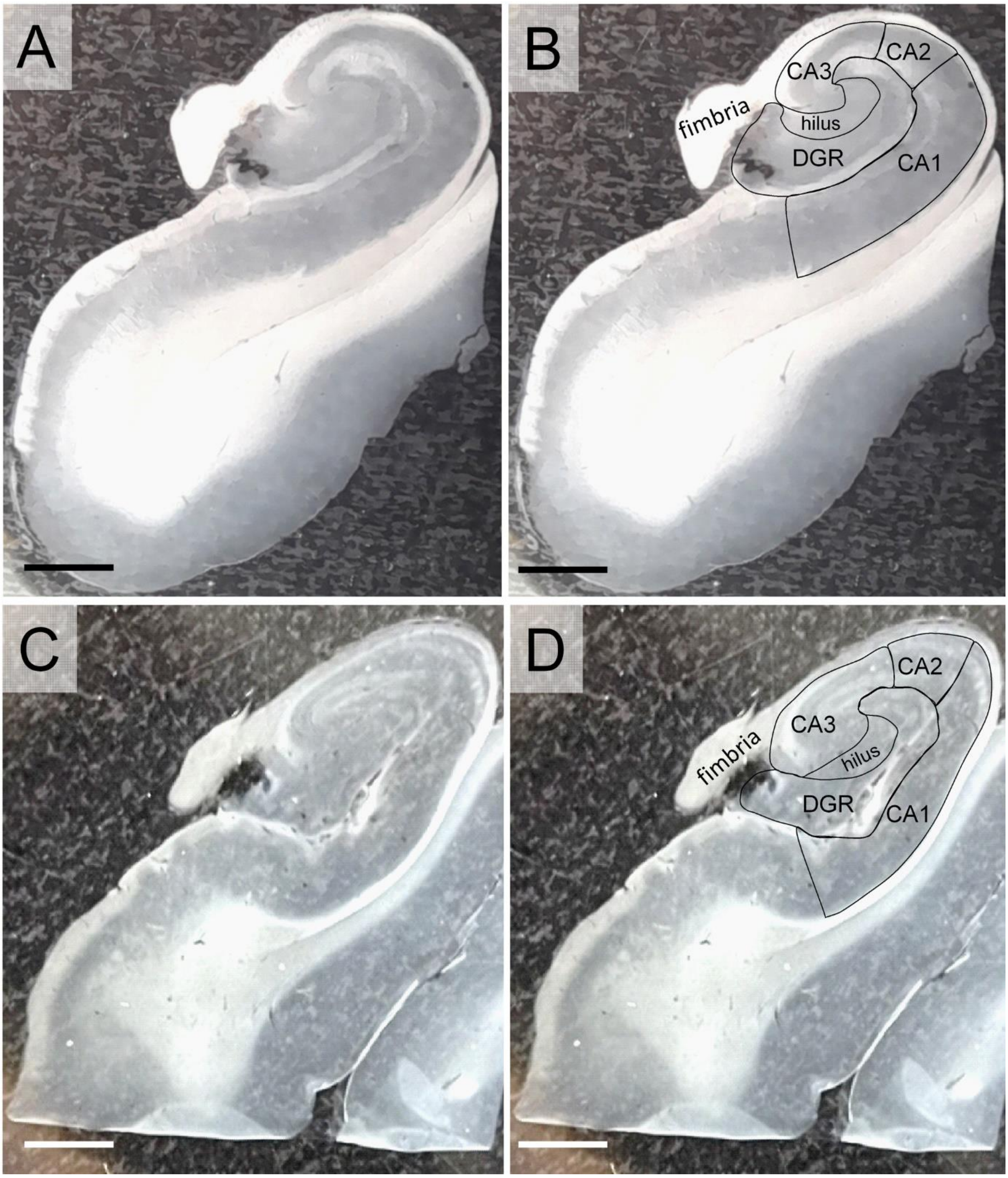

Supplementary Figure 7.

Representative native free-floating sections of the human hippocampal formation with adjacent temporal white and gray matter areas. The photographed sections were cut from mediobasal temporal tissue blocks of donor brains No. SKO29 (A) and TBSKO1 (C). Panel A represents a hippocampal formation coronal section plane that corresponds to the 54th coronal section in

the Allen Human Brain reference atlas shown at this link: (https://atlas.brain-map.org/atlas?atlas=265297126#atlas=265297126&plate=112360820&structure=10247&x=38362.66796875&y=63205.33203125&zoom=-6&resolution=62.05&z=3). The Panel C images show a plane that corresponds to the plane No 57 in the above referenced atlas that can be observed here: (https://atlas.brain-map.org/atlas?atlas=265297126#atlas=265297126&plate=112360771&structure=10247&x=38362.66796875&y=63203.19921875&zoom=-6&resolution=62.18&z=3). Panel B and D show copies of images in A and C, respectively. In B and D, the anatomical boundaries of the hippocampal areas including the cornu Ammonis (CA) 1 area (CA1), CA area 2 (CA2), CA area 3 (CA3), the dentate gyrus (DGR) and the hilus were indicated. Fimbria: fimbria hippocampi. Mossy cells indicated in Figure 7. were found in the hilus of similar serial sections of the same tissue blocks. Scale bars: 3mm.

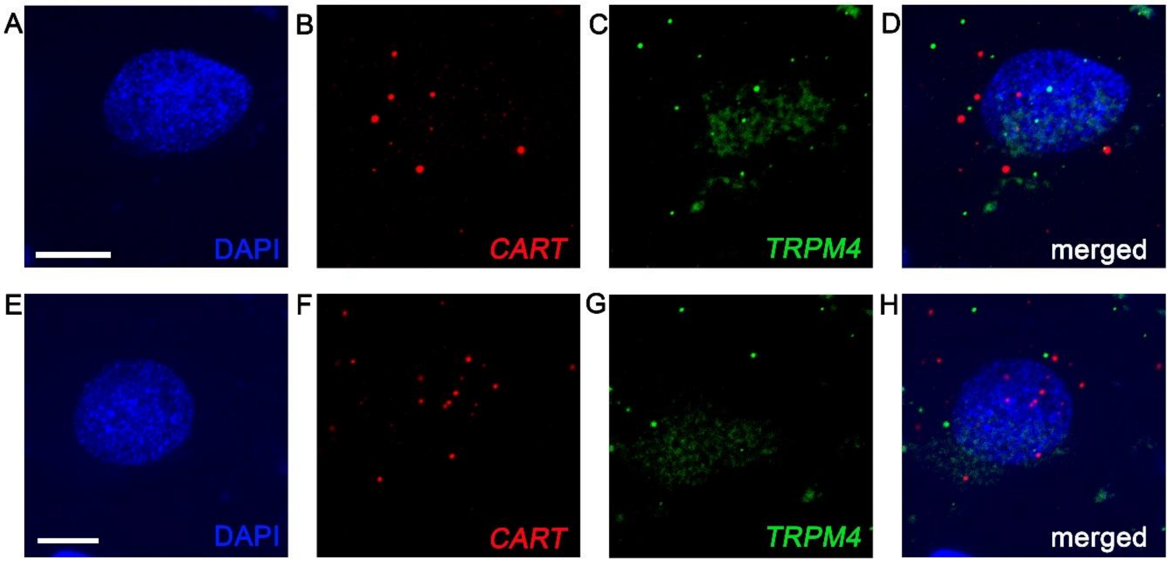

Supplementary Figure 8.

Representative fluorescence images taken from human hippocampi (different sample than showed in Figure 7) showing *TRPM4* mRNA transcripts (green) co-expressed with the cocaine- and amphetamine-regulated transcript (*CART*, red) mossy cell marker. Nuclear counterstaining was performed with 4′,6- diamidino-2-phenylindole (DAPI, blue). Please note, that beyond the dot-like specific RNAscope signal puncta, a weak blurry signal is recognizable predominantly in the green channel that is caused by autofluorescence due to lipofuscin accumulation often seen in aged human neurons. Scalle bar is 5 µm.

Supplementary Table 1. Characteristics of human brain samples used for RNAscope *in situ* hybridization.

Case code	Gender	Age	*Post mortem* time (min)
TBSKO1	Female	61 Years	300
SKO29	Female	69 Years	212

Supplementary Table 2.

Summary of statistics:

	n number	mean	SD	test	p value/t value/DF
**Figure 1 D**	saline= 9<br>Meclo= 8	14.92<br>18.38	7.06<br>10.73	Two sample<br>T-test	0.44029/-0.79/15
**Figure 1 E**	saline= 9<br>Meclo= 8	34.59<br>51.28	7.09<br>14.22	Two sample<br>T-test	0.00703/-3.11/15
**Figure 1 F** theta	saline= 9<br>Meclo= 6	11.25<br>11.77	55.86<br>12.34	Two sample<br>T-test	0.91/-0.11/13
**Figure 1 F** alpha	saline= 9<br>Meclo= 6	2.88<br>3.58	1.57<br>3.43	Two sample<br>T-test	0.59/-0.53/13
**Figure 1 F** beta	saline= 9<br>Meclo= 6	0.88<br>1.2	0.45<br>0.77	Two sample<br>T-test	0.32/-1.02/13
**Figure 1 F** gamma	saline= 9<br>Meclo= 6	0.16<br>0.16	0.05<br>0.06	Two sample<br>T-test	0.89/-0.13/13
**Figure 2 C**	saline=9<br>Meclo=8	7699.88889<br>4259.625	4573.22814<br>2950.3284	Mann-<br>Whitney<br>Test	0.04854/-1.26/15
**Figure 2 D**	saline= 9<br>Meclo=8	0.84889<br>1.84125	0.34101<br>1.35804	Two sample<br>T-test	0.04051/-2.12/15
**Figure 2 E**	saline= 9<br>Meclo=8	34.46<br>18.18	16.27<br>13.10	Two sample<br>T-test	0.03931/2.25/15

**Figure 2 F**	saline= 9<br>Meclo= 8	0.461<br>0.59	0.13918<br>0.26812	Two sample<br>T-test	0.22434/-1.26/15
**Figure 2 G**	saline= 9<br>Meclo= 8	25.88<br>22.25	10.0305<br>16.833	Two sample<br>T-test	0.5909/0.54/15
**Figure 3 C**	saline =8<br>Meclo = 9	14<br>20	4.37<br>7.01	Two sample<br>T-test	0.549/-2.08/15
**Figure 3 D**	saline =8<br>Meclo = 9	25.64<br>17.57	14.3<br>4.89	Two sample<br>T-test	0.1312/1.5/15
**Figure 3 E**	saline =8<br>Meclo = 9	30.46<br>21.72	14.54<br>9.13	Two sample<br>T-test	0.153/1.5/15
**Figure 3 F**	saline =8<br>Meclo = 9	19.87<br>22.74	10.71<br>7.38	Two sample<br>T-test	0.525/-0.64/15
**Figure 3 G**	saline =8<br>Meclo = 9	1.19<br>0.84	0.85<br>0.41	Two sample<br>T-test	0.283/1.11/15
**Figure 3 H**	saline =8<br>Meclo = 9	0.466<br>0.513	0.201<br>0.151	Two sample<br>T-test	0.59/-0.54/15
**Figure 3 I**	saline =6<br>Meclo =6	5677.25<br>6208.22	2932.79<br>2500.77	Two sample<br>T-test	0.69/-0.4/15
**Figure 4 D**	Saline=8<br>Saline+KA =9<br>Meclo+KA =8	73.12<br>50.7<br>51.47	8.14<br>7.82<br>11.39	One Way<br>ANOVA,<br>Tukey's *post hoc*	Saline vs saline+KA<br>0.00015<br>Saline vs Meclo+KA<br>0.0003
**Figure 4 E**	Saline=8<br>saline = 19<br>Meclo =17	82<br>58<br>91.93	9.41<br>17.28<br>13.81	One Way<br>ANOVA,<br>Tukey's *post hoc*	Saline vs saline+KA<br>0.0053<br>Saline+KA vs<br>Meclo+KA 0.00016
**Figure 5 D**	$\text{Saline}_{\text{central}}$ =10<br>$\text{Saline}_{\text{ventral}}$ =10	7.99<br>9.55	1.04929<br>1.57215	Two sample<br>T-test	0.01773/-2.6/18
**Figure 6 B**	ACSF = 10<br>Meclo=10	0.894<br>0.415	1.12905<br>0.57214	Wilcoxon<br>Signed<br>Ranks Test	0.04883

**Figure 6 F**	ACSF = 11<br>Meclo = 11	1.93<br>1.755	0.28<br>0.31	Paired Sample T-test	0.0016/4.14/11
**Figure 6 H**	ACSF=11<br>Hyperexcitable=11<br>ACSF=11<br>Hypererxcitable + Meclo=11	0.13909<br>1.78545<br>0.06<br>0.62818	0.2723<br>1.55403<br>0.08798<br>0.69329	One Way ANOVA, Tukey's *post hoc*	1) ACSF vs Hyperexcitable = 0.00034/9.35/43<br>2) Hyperexcitable vs Hyperexcitable+Meclo = 0.0158/9.35/43
**Figure 6 J**	ACSF=11<br>Hyperexcitable=11<br>ACSF=10<br>Hypererxcitable +<br>Meclo=10	0.54364<br>6.05727<br><br>0.696<br>4.361	0.35328<br>3.61256<br><br>0.61833<br>2.91711	One Way ANOVA, Tukey's *post hoc*	1) $ACSF_{SE}$ vs SE = 0.00001/14.3/41<br>2) $ACSF_{SE+Meclo}$ vs SE+Meclo = 0.00364/14.3/41<br>3) SE vs SE+Meclo= 0.3667/14.3/41
**Suppl. Figure 4. A**	Saline=6<br>Meclo=7	1183.33<br>501.71	1827.61<br>1198.83	Mann-Whitney Test	0.026/2/31
**Suppl. Figure 4. B**	Saline=6<br>Meclo=7	82.5<br>4.71	143.96<br>12.47	Mann-Whitney Test	0.061/1.69/28
**Suppl. Figure 4. C**	Saline=6<br>Meclo=7	2.83<br>0.14	2.99<br>0.37	Mann-Whitney Test	0.041/1.78/28.5
**Suppl. Figure 6. A**	Saline=11<br>Meclo=11	-64.45<br>-65.35	11.8<br>12.8	Paired Sample T-test	0.71/0.37/10

**Suppl. Figure 6. B**	Saline=11<br>Meclo=11	100.81<br>96.9	48.07<br>37.11	Paired Sample T-test	0.64/0.46/10
**Suppl. Figure 6. C**	Saline=11<br>Meclo=11	41.09<br>41.18	2.8<br>3.6	Paired Sample T-test	0.89/-0.13/10
**Suppl. Figure 6. D**	Saline=11<br>Meclo=11	67.09<br>66.38	5.76<br>5.91	Paired Sample T-test	0.19/1.37.10
**Suppl. Figure 5. A**	Saline=8<br>Meclo=8	0.19<br>0.31	0.14<br>0.38	Wilcoxon Signed Ranks Test	0.32
**Suppl. Figure 5. B**	Saline=8<br>Meclo=8	1.92<br>1.91	0.09<br>0.09	Paired Sample T-test	0.9/0.13/6
